# Discovery and characterisation of terpenoid biosynthesis enzymes from *Daphniphyllum macropodum*

**DOI:** 10.1186/s12870-025-06421-0

**Published:** 2025-04-16

**Authors:** Kaouthar Eljounaidi, Caragh B. Whitehead, Emily Radley, Marissa H. Petrou, Katherine Newling, Sally James, Benjamin R. Lichman

**Affiliations:** 1 Centre for Novel Agricultural Products, Department of Biology, University of York, York, YO10 5DD UK; 2 Biosciences Technology Facility, Department of Biology, University of York, York, UK; 3 Department of Chemistry, Manchester Institute of Biotechnology, The University of Manchester, 131 Princess Street, Manchester, M1 7DN UK

**Keywords:** Terpenoids, Biosynthesis, *Daphniphyllum macropodum*, Terpene synthases, Specialised metabolism, *Nicotiana benthamiana*

## Abstract

Terpene synthase (TPS) enzymes are known to be pivotal in the biosynthesis of terpenoids in plants and microbes. These enzymes catalyse the transformation of ubiquitous acyclic prenyl diphosphate substrates into diverse hydrocarbon skeletons. Mining TPS genes from the genomes and transcriptomes of diverse taxa of plants and microbes is a powerful tool for the discovery of new enzymes and new chemistries. In this work, we used a combination of long-read and short-read RNA sequencing technologies to develop a high-quality transcriptomic data set for *Daphniphyllum macropodum*, a poorly studied plant rich in unique terpene derived metabolites. Through mining the transcriptome, we identified different terpene-related enzymes, including prenyl transferases (PTs), triterpene cyclases (TTCs), and terpene synthases (TPSs). These were expressed in a *Nicotiana benthamiana* system together with rate-limiting enzymes HMGR or DXS to boost the metabolic flux. The products were then analysed using GC-MS. We have identified four monoterpene synthases producing diverse linear, monocyclic and bicyclic monoterpenes including linalool, limonene, geraniol, and pinene. We also identified four sesquiterpene synthases, one producing caryophyllene, one α-guaiene, and two producing a blend of sesquiterpenes. In addition, we demonstrated the activity of two triterpene cyclases both forming cycloartenol, as well as a geranylgeranyl diphosphate synthase (GGPP synthase). Together, these findings expand the known chemical space of *D. macropodum* terpenoid metabolism and enable further investigations of terpenoids in this under-explored plant species.

**Supplementary Information:**

The online version contains supplementary material available at 10.1186/s12870-025-06421-0.

## Background

Terpenoids are the largest and most diverse group of natural products produced by plants and microbes. These compounds are known for their wide range of biological activities [1]. They derive from two C5 isoprene precursors: isopentenyl diphosphate (IPP) and dimethylallyl diphosphate (DMAPP). In plants IPP and DMAPP are synthesised via mevalonate (MVA) and methylerythritol (MEP) pathways. Through the catalytic action of prenyltransferases (PTs), these precursors are sequentially condensed to form prenyl pyrophosphate (prenyl-PP) substrates such as geranyl pyrophosphate (GPP), farnesyl pyrophosphate (FPP), and geranylgeranyl pyrophosphate (GGPP). Terpene synthases (TPSs) then convert these substrates into a vast array of structurally diverse terpenoid scaffolds. The classification of terpenoids is based on the number of isoprene units: monoterpenes (C10), sesquiterpenes (C15), diterpenes (C20) and triterpene (C30). For the biosynthesis of triterpenes, triterpene cyclases (TTCs), also known as oxidosqualene cyclases, catalyse the cyclisation of 2,3-oxidosqualene, leading to a wide variety of triterpene structures [2].

*Daphniphyllum macropodum* is an evergreen shrub native to East Asia, particularly found in Japan, Korea, and China. It belongs to the Daphniphyllaceae family and is known for its glossy, dark green leaves and distinctive red petioles [3, 4]. *D. macropodum* is often grown as an ornamental plant due to its attractive foliage and has been utilised in traditional Chinese medicine to treat diverse conditions such as asthma and rheumatism [5]. Notably, *Daphniphyllum* species are recognised for producing structurally diverse and complex terpene alkaloids, characterised by intricate stereochemical frameworks and polycyclic cage-like structures [4, 6]. *D. macropodum* is a non-model plant that occupies an underexplored evolutionary niche, with limited genetic information currently available. It is a member of the Saxifragales, a relatively small but highly morphologically diverse angiosperm order [7].

In this study, a high-quality transcriptome of *D. macropodum* was generated using both long and short-read sequencing technologies. Following functional annotation, new genes including three TTCs and ten TPSs were identified, and their gene expression levels were analysed. Comparison of TPSs expression across different tissues—leaf buds, male and female flowers, and immature leaves—revealed distinct patterns, especially between male and female flowers.

Through heterologous expression in *N. benthamiana*, key rate-limiting enzymes from *D. macropodum* terpene precursor biosynthesis were characterised. These genes were then co-expressed in *N. benthamiana* with various candidate genes to assess their catalytic activity. Through this analysis new monoTPSs, sesquiTPSs, and TTCs were characterised. This included two different linalool synthases that produce the (*R*)-linalool and (*S*)-linalool enantiomers, as well as several multiproduct TPSs generating a variety of monocyclic and bicyclic terpenoids. In addition, a PT from *Daphniphyllum* was identified as geranylgeranyl pyrophosphate synthase (GGPPS). These findings underscore the diversity of terpene metabolism in *D. macropodum*, laying the groundwork for further studies to deepen our understanding of terpenoid evolution and biosynthesis in *D. macropodum*, Daphniphyllaceae and the Saxifragales.

## Methods

### Chemicals and plant materials

All the authentic standards and substrates used in this study were purchased from Sigma-Aldrich (http://www.sigmaaldrich.com); including valencene, isoprene, caryophyllene, limonene, α-pinene, R-linalool, linalool, geranyl linalool, squalane, farnesyl pyrophosphate ammonium salt and cyclohexanone. *Nicotiana benthamiana* plants were grown on F2 + S seed and modular compost (Levingston Advance) and kept and maintained for 4 to 5 weeks in the greenhouse. The growing conditions were 16-hour photoperiod and a temperature of 22 °C during the day and 20 °C during the night.

### RNAseq and transcriptome assembly

Plant material was obtained from seven plants growing in the UK (Table S1). Tissues (leaf buds, male and female flowers and immature leaves) were harvested onto dry ice (Table S2). These tissues were collected due to their accessibility and ease of harvest from the available trees/plants. DNA-depleted RNA was isolated using a Direct-Zol RNA miniprep kit (Zymo Research, Irvine, CA, USA). RNA was assessed using the Agilent Tapestation system with RNA nano screentape. For short read sequencing, library preparation was performed from 200 ng high quality total RNA input using the NEBNext Ultra II Directional Library prep kit for Illumina in conjunction with the NEBNext Poly(A) mRNA Magnetic Isolation Module and unique dual index set (New England Biolabs, Ipswich, MA, USA), according to the manufacturer’s instructions. Libraries were pooled at equimolar ratios and sent for paired end 150 base sequencing at Novogene (UK) Company Ltd. on a NovaSeq 6000 instrument (Illumina, San Diego, CA, USA), with 10 million reads per sample on average.

For full length cDNA sequencing, libraries were prepared from 100 ng total RNA per plant with the Oxford Nanopore Technologies (ONT) PCR cDNA barcoding library prep kit SQK-PCB109, including a 12 cycle PCR amplification of full-length cDNA, with unique barcodes for each sample. Then, equal quantities of cDNA from each plant were pooled into final 100 fmol cDNA pool for adapter ligation and loading onto ONT R9.4.1 flow cells and sequencing on an ONT promethION running MinKNOW (v20.06.9). Basecalling and barcode demultiplexing were performed using guppy basecaller (v4.0.11) and quality-checked with PycoQC. Reads from all samples were then combined for a *de novo* transcriptome assembly using Rattle. The resulting assembly was polished with Medaka (https://github.com/nanoporetech/medaka) using the ONT reads, followed by Pilon polishing (x3) using combined Illumina short reads. The Illumina reads were quality checked with FastQC, trimmed with Cutadapt, and any ribosomal RNA removed with BBSplit. Transcripts were filtered using the Perl script tr2aacds.pl from Evigene [8] and annotated with Transdecoder to identify open reading frames (https://github.com/TransDecoder/TransDecoder). Salmon (v1.1.0) [9] (parameters “--validateMappings -l ISR --seqBias --gcBias --numBootstraps 100”) was used to map reads to the transcriptome, and expression was calculated with Salmon, run via the Sleuth R package [10]. Quality assessment of the transcriptome was performed with BUSCO (v5.4.3) using the embryophyta_odb10 database [11].

### Cloning of candidate genes

Predicted peptides were annotated using InterProScan (default settings), for domain annotations, and the curated SwissProt database (BlastP, E-value < 0.05). Relevant terpenoid genes were identified using a combination of InterPro accessions (e.g. IPR005630 for terpene synthases), SwissProt GO terms (e.g. terpenoid biosynthetic process [GO:0016114]) and SwissProt protein name annotations (e.g. “Probable terpene synthase”). Shortlisted genes were also validated by blast matches to *Arabidopsis* proteins (TAIR10 representative peptides, https://www.arabidopsis.org/, BlastP, E-value < 0.05). To amplify the open reading frame of the gene of interest, primers for cloning (from Integrated DNA Technologies (IDT)) were designed with overlaps having homology with the plant expression vector pHREAC [12] (Table S3). All primers were designed to obtain the entire open reading frame of the genes except for the primers for HMGR1 and HMGR2 which respectively lack the initial 492 and 510 nucleotides, mimicking the truncation of *Avena strigosa* HMGR in Reed et al., (2017) [13]. The truncation of this region usually results in a feedback-insensitive protein and enhances yields of MVA end products. *D. macropodum* genes were amplified from *D. macropodum* young leaf cDNA. Linalool synthase AtLS (GenBank accession: AF497485.1) and geranyl linalool synthase AtGLS (GenBank accession: AEE33784.1) were both amplified from *Arabidopsis thaliana* young leaf cDNA. Valencene synthase from *Callitropsis nootkatensis* CnVS (GenBank accession: JX040471) was synthesised by Twist Bioscience (San Francisco, CA, USA). (Table S4). The full-length genes were amplified using Platinum™ SuperFi II Green PCR Master Mix (Thermo Fisher Scientific). The DNA products were then inserted using In-Fusion (Takara Bio Inc.) reaction into pHREAC vector and the In-Fusion reaction mixtures were transformed into Stellar *Escherichia coli* (Takara Bio Inc.). Plasmids were then extracted using the QIAprep Spin Miniprep Kit (Qiagen) and verified for sequence integrity by Sanger sequencing (Eurofins Genomics).

### Heterologous expression in *N. benthamiana*

The plasmids were transformed into competent *Agrobacterium tumefaciens* strain LBA4404 cells using heat-shock method. The recombinant *A. tumefaciens* strains were grown at 28 °C for 24 h at 220 rpm in liquid LB media containing streptomycin (50 mg L^− 1^) and kanamycin (50 mg L^− 1^). After centrifugation at 4000 x g at 20 °C for 20 min, cells were resuspended in 10 mM MES buffer containing 10 mM MgCl_2_ and 100 µM acetosyringone to a final OD600 of 0.5 and left to incubate at room temperature for 2 h at 50 rpm. The abaxial side of leaves of 4-week-old *N. benthamiana* plants was injected with the prepared *A. tumefaciens* strains using a 1 mL syringe in triplicate for each gene. For co-infiltration, equal volumes of different recombinant *Agrobacterium* strains were mixed. The Agro-infiltrated plants were left to grow for 5 days before harvesting for analysis.

### Metabolite extractions

Terpenoid extraction from the agroinfiltrated leaves of *N. benthamiana* required different methods depending on the subclass of the expected products. For the extraction of monoterpenes, sesquiterpenes and diterpenes, ethyl acetate was used; this included the products of CnVS, TPSg2, TPSa1, TPSa2, TPSa3 and PT3 + AtGLS. To 300 mg of ground tissue 300 µL volume of analytical grade ethyl acetate was added containing 0.05 mM of cyclohexanone as an internal standard. After an hour of shaking at 800 rpm at room temperature the samples were centrifuged at 21,000 g for 10 min. Then 100 µL of the upper phase were transferred to a 1.1 mL tapered glass vial (Thermofisher Scientific, Germany) and crimp sealed, before the GC-MS analysis.

For the extraction of triterpenes, a saponification step was used, this included the extraction of the products of TTC1 and TTC2. To 300 mg of the harvested and ground *N. benthamiana* tissue 500 µL of a saponification solution (EtOH: H_2_O: KOH, 9:1:1, v: v:w) with squalane (0.05 mM) as the internal standard was added. After 2 h of incubation at 65 °C with intermittent agitation 200 µL of H_2_O and 500 µL hexane were added before vortexing (2 × 10s) and transferring 200 µL of the upper hexane phase to a new vial. The samples were then dried and derivatized with 60 µL pyridine, 40 µL N-methyl-N-(trimethylsilyl)trifluoroacetamide (MTFA) and 1% trimethylsilyl chloride at 50 °C for 1 h.

For the analysis of other volatile chemicals such as monoterpenes and isoprene HS-SPME (headspace solid phase micro-extraction) method was used, this included the products for TPSb1, TPSb2, TPSg3, TPSg4 and TPSb3. For this analysis and on day 4 after the agroinfiltration, the agroinfiltrated *N. benthamiana* leaves were cut with their petioles and placed in headspace vials containing 3 mL of water. The vials were sealed well using a screw cap and left for additional 24 h in the greenhouse before performing the SPME analysis on the next day. The analysis of volatiles (HS-SPME) of *Daphniphyllum macropodum* tissues was performed as follows. Tissues of leaves and fruits from *D. macropodum* were harvested and ground with liquid nitrogen. Then, 500 mg of each sample were placed in a 20 mL glass vial containing 2 mL of tartrate extraction buffer (5 g L^− 1^ tartaric acid, 2 g L^− 1^ ascorbic acid, 8 mg L^− 1^ sodium azide and 250 g L^− 1^ NaCl). The vials were sealed with screw caps and then analysed. For the measurements of terpenoids in the extracts, the peak area of the target chemical was normalised based on the corresponding internal standard and tissue weight. For each condition three independent biological replicates were used. Statistical significance was assessed using a one-tailed Student’s t-test with an assumption of unequal variance through an R script.

### GC-MS analysis

Agilent 7890 A gas chromatograph (Agilent Technologies) interfaced to an Agilent 5975 C MSD (Agilent Technologies) was used for the GC-MS analyses of all the samples. For all the direct injection analyses the separation was performed in splitless mode on a Zebron ZB5-HT-INFERNO column (length: 30 m; diameter: 250 μm; Phenomenex) interfaced with auxiliary transfer line (1.5 m, 150 μm). Helium was used as a mobile phase at a constant flow of 1.4 mL/min. 2 µL of each sample were injected. The oven temperature was set to 50 °C for 3 min post-injection, increased to 150 °C at 3 °C/min and then to 280 °C at 10 °C/min, where it was held for 10 min. The mass scan range was 50–550 m/z. For the HS-SPME analysis 50/30 µm gray divinylbenzene/carboxen/ polydimethylsiloxane (DVB/CAR/PDMS) fiber was used. Prior to the extraction, the fiber was preconditioned according to the manufacturer’s instructions. Sample vials were pre-incubated for 10 min at 60 °C and agitated at 250 rpm. The extractions were carried out for 45 min at 60 °C and the desorption was at 250 °C for 180 s. The separation of volatiles was achieved with the same GC-MS system described above equipped with a chiral GC column beta-DEX 225 (length: 30 m; diameter: 250 μm, fused silica capillary column; Supelco). Helium was used as the carrier gas at a flow rate of 1.0 mL/min. The oven temperature was held at 50 °C for 1 min, increased to 150 °C at 2 °C/min, held for 2 min and then increased 50 °C /min to 220 °C then held for 2 min. The mass scan range was 50–550 m/z.

### Heterologous expression in *E. coli* and enzyme assays

TPSa1 and TPSa3 were synthesised by Twist Bioscience (San Francisco, CA, USA) and optimised for *E. coli* codon usage. Both genes were inserted into the pET28a plasmid with an N-terminal hexhistidine tag. Furthermore, truncations of 19 and 18 amino acids, respectively, were made at the 5’ end of the sequences to enhance their solubility. SoluBL21(Genlantis) *E. coli* strains expressing TPSa1 and TPSa3 were grown at 37 °C in 2X YT media with 50 µg/mL kanamycin. Protein expression was induced at OD600 between 0.5 and 0.7 with 0.5 mM IPTG and an overnight incubation at 18 °C. After 16 h cells were pelleted and resuspended in Lysis buffer (0.2 mg/mL lysozyme, 50 mM Tris-HCL, 50 mM glycine, 5% v/v glycerol, 0.5 M NaCl, 20 mM imidazole, pH 8) with Complete Mini EDTA-free Protease Inhibitor Cocktail (Roche Diagnostics). The sample was then lysed with cell press at 25 kPSI followed by high-speed centrifugation (35000 g, 20 min) to remove cell debris. The lysate was mixed with Co-NTA agarose resin (BioServUK, UK) and incubated at 4 °C with gentle shaking. After 1 h, the resin was washed once with buffer A (50 mM Tris-HCL, 50 mM glycine, 5% v/v glycerol, 0.5 M NaCl, 20 mM imidazole, pH 8) and once with buffer B (50 mM Tris-HCL, 50 mM glycine, 5% v/v glycerol, 0.5 M NaCl, 40 mM imidazole, pH 8). Proteins were then eluted with elution buffer (50 mM Tris-HCL, 50 mM glycine, 5% v/v glycerol, 0.5 M NaCl, 500 mM imidazole, pH 8). The buffer was exchanged using PD10 desalting columns (Merck Life Sciences, UK), and the proteins were subsequently concentrated with Vivaspin centrifugal concentrators (Sartorius, Germany). Protein purity and quantity were verified through SDS-PAGE gel electrophoresis and spectrophotometric analysis at 280 nm. The purified proteins (0.5 µM) were assayed in 50 mM Tris-HCl (pH 7.5), 10 mM MgCl2, 1 mM DTT, 5% glycerol, and 58 µM FPP in a total volume of 100 µL. After 2 h of shaking (500 rpm) at 30 °C the reactions were stopped by the addition of 100 µL of 4 M NaOH and 1 M EDTA. The metabolites were finally extracted with 200 µL of ethyl acetate and analysed with the GC-MS as described above.

### Phylogenetic analysis

For the phylogenetic analysis of monoterpene and sesquiterpene synthases, full-length amino acid sequences of various terpene synthases were collected from UniProt using BLASTP, with *Daphniphyllum macropodum* genes as queries. The collected 188 sequences were aligned with MAFFT multiple protein alignment [14], using geneious prime software 2023 (https://www.geneious.com). This alignment was then used to infer maximum likelihood tree via IQ-Tree 2 [15] with ModelFinder [16] and ultrafast bootstraps (UFBoot, X1000) [17], and SH-aLRT supports (X1000) [18]. The result tree was visualised using iTOL 6.3.2 [19]. For the phylogenetic analysis of farnesyl pyrophosphate synthases, FPPS1 and FPPS2 sequences were used as queries in BLASTP searches against the JGI Phytozome 12, the China National GeneBank and 1000 Plants project (OneKP) databases, to identify homologs. The collected 408 sequences were then used to infer the phylogenetic tree as described above. For the TTC tree, a multiple sequence alignment was performed using MAFFT and phylogeny inferred using IQ-Tree as described above. For the phylogenetic analysis of geranyl(geranyl) pyrophosphate synthases (G[G]PPSs) sequences were obtained from Figure S1 of Song et al. [20], alongside sequences from *D. macropodum*; a multiple sequence alignment was performed using MAFFT and phylogeny inferred using iQTree as described above. Clades were labelled as per Song et al. [20].

## Results

### Identification and characterisation of MEP/MVA pathway genes

**Fig. 1:**
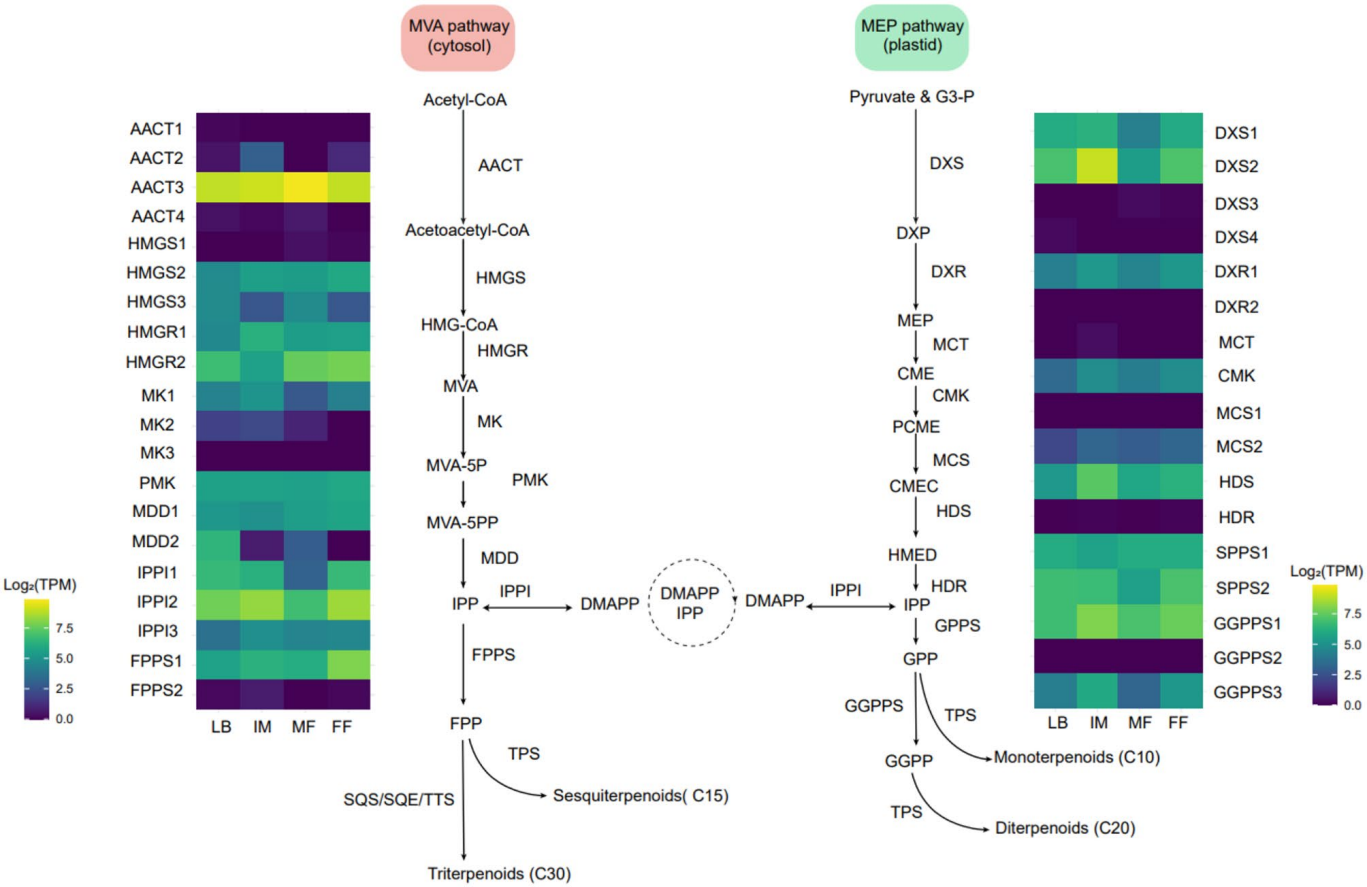
Identification of Mevalonate/MEP pathway genes. Heatmaps showing the log2 values of the TPM (transcripts per million) of each gene involved in mevalonate (MVA) and methylerythritol phosphate (MEP) pathways in different plant tissue types. Abbreviations for tissues: LB, leaf buds; IM, immature leaves; MF, male flowers; FF, female flowers. Abbreviations for the genes are listed in Table S5. Additional abbreviations are listed here: G-3P, glyceraldehyde 3-phosphate, HMG-CoA, 3-hydroxy-3-methylglutaryl CoA; MVA, Mevalonate; MVA-5P, Mevalonate 5-phosphate; MVA-5PP, Mevalonate 5-diphosphate; IPP, Isopentenyl diphosphate; DMAPP, Dimethylallyl diphosphate; GPP, Geranyl diphosphate; FPP, Farnesyl diphosphate; GGPP: Geranylgeranyl diphosphate. DXP, 1-deoxy-D-xylulose 5-phosphate; MEP, 2-C-methyl-D-erythritol 4-phosphate; CME, 4-(cytidine 5’-diphospho)-2-C-methyl-D-erythritol; PCME, 4-(cytidine 5’-diphospho)-2-C- methyl-D-erythritol 2-phosphate; CMEC, 2-C-methyl-D-erythritol 2,4-cyclodiphosphate; HMED, (E)-4-Hydroxy-3-methyl-but-2-enyl pyrophosphate; SQS, squalene synthase; SQE. Squalene epoxidase; TTS, triterpene synthase

A *de novo* transcriptome assembly for *D. macropodum* was generated by combining two RNA sequencing technologies: Illumina (paired-end short reads) and Oxford Nanopore sequencing (Nanopore PromethION, long reads). The quality of the *de novo* assembly (i.e. no reference genome) was judged to be high based on the identification of near universal single-copy orthologs. Of 1614 queried orthologs, 1527 were found in the assembly complete (94.6%), 24 fragmented (1.5%) and only 63 were missing (3.9%). From open reading frame prediction on the 42,433 transcripts, we predicted 53,205 peptides, of which 41,584 had domain annotations (from InterProScan). Short-read sequencing libraries from multiple *D. macropodum* tissues were mapped to the transcriptome to quantify relative gene expression. The tissues used were leaf buds, male and female flowers and immature leaves, determined by a combination of tissue availability and RNA quality. All MVA and MEP pathway genes in *D. macropodum* (Table S5) were annotated, and their full-length sequences were identified, demonstrating the high quality and completeness of the transcriptome. Tissue-specific expression TPM (transcripts per million) of each gene was log2 normalised to effectively ascertain the fold change before being plotted as a heat map. Our analysis showed that the variability in gene expression in these pathways was evident at the level of individual genes, among paralogs, and across different tissue types (Fig. 1). Among MEP pathway genes, DXS2 showed the highest transcript abundance compared with the rest of the pathway genes. Within the MVA pathway, AACT3, IPPI2 and HMGR2 displayed the highest expression levels. Further analysis revealed that the difference of expression levels between paralogs was quite noticeable, for example, HMGR2 was more expressed overall in all tissues compared with HMGR1. Additionally, DXS2 was the most expressed transcript among the four annotated paralogs (DXS1, DXS2, DXS3 and DXS4).

**Fig. 2:**
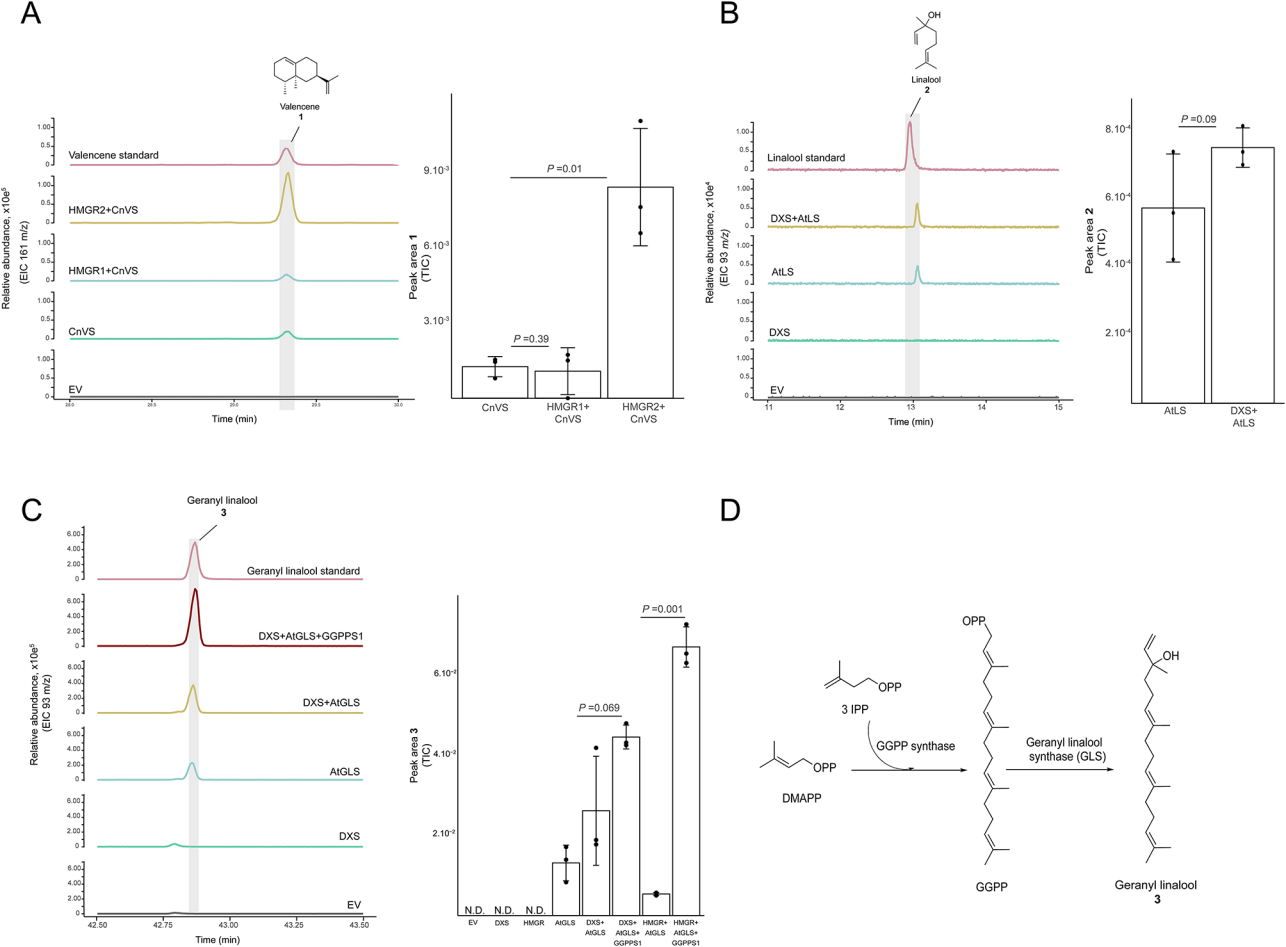
Characterisation of Mevalonate and MEP pathway genes. **(A)** GC-MS analysis of *Nicotiana benthamiana* extracts expressing truncated versions of HMGR 1 or 2 with valencene synthase of *Callitropsis Nootkatensis* (CnVS). On the left the GC-MS traces are shown as extracted ion chromatograms (EIC m/z 161), the product ID was confirmed by comparison with an authentic valencene standard. On the right, peak areas of valencene **1** in the investigated tissues are shown. **(B)** GC-MS analysis of *Nicotiana benthamiana* extracts expressing DXS with Linalool synthase of *Arabidopsis thaliana*. On the left the GC-MS traces are shown as extracted ion chromatograms (EIC m/z 93), the product ID was confirmed by comparison with an authentic linalool standard. On the right, the peak areas of linalool **2** in the investigated tissues are being shown. **(C)** GC-MS analysis of *N. benthamiana* extracts expressing PT3 with AtGLS. The GC-MS traces are presented as extracted ion chromatograms (EIC 93 m/z); geranyl linalool was confirmed by comparison with an authentic standard. On the right, comparison of peak areas of geranyl linalool **3** in the extracts of plants expressing different combinations of genes to characterise the activity of PT3. These combinations are DXS + AtGLS VS DXS + AtGLS + PT3 and then HMGR + AtGLS VS HMGR + AtGLS + PT3. **(D)** Biosynthetic route to geranyl linalool **3**. For A, B, and C, three independent biological replicates were used for each condition. data are mean ± s.d. of the three replicates. Statistical significance was assessed using a one-tailed Student’s t-test with an assumption of unequal variance

Co-expression of truncated and feedback-insensitive 3-hydroxy-3-methylglutaryl CoA reductase (tHMGR) from various plants was shown to increase the production of terpenes in *N. benthamiana* [21]. The effects of the inclusion of a truncated version of the two paralogs of *D. macropodum* HMGRs: HMGR1 and HMGR2 on valencene **1** production, was evaluated using valencene synthase from *Callitropsis nootkatensis* (CnVS) (Fig. 2A). The addition of HMGR1 had no impact on valencene **1** accumulation, however the co-expression of HMGR2 with CnVS increased valencene **1** production by ~ 7-fold, demonstrating that HMGR2 significantly boosts terpene production *in planta*. In all the subsequent co-expression experiments the HMGR variant that was used to boost the metabolic flow in *N. benthamiana* was HMGR2.

Another rate limiting enzyme in terpenoid biosynthesis is 1-deoxy-*D*-xylulose-5-phosphate synthase (DXS). It is a key enzyme catalysing the first committed step of MEP pathway, converting pyruvate and glyceraldehyde-3-phosphate into 1-deoxy-D-xylulose-5-phosphate (DXP) (Fig. 2B). Overexpression of DXS-encoding genes from various plant species in *N. benthamiana* has consistently resulted in an increased production of terpenoid [21]. Among the four annotated DXSs in *D. macropodum* only DXS2, which was the most expressed paralog as mentioned above, was isolated and amplified. When DXS2 was co-expressed with *Arabidopsis thaliana* linalool synthase an overall increase in linalool **2** accumulation was observed (Fig. 2B).

### Prenyltransferases (PTs) from *D. macropodum*

We initially found seven genes corresponding to canonical terpenoid forming prenyltransferases (i.e. geranyl/farnesyl pyrophosphate synthases). To determine their identity and evolutionary origin more precisely than using best-match blast searches, we constructed a prenyltransferase phylogeny, based on a recent investigation of the G(G)PPS plant gene family (Figure S1) [20]. We found two sequences corresponding to solanesyl pyrophosphate synthases (SPPSs), which are sometimes erroneously annotated as geranyl pyrophosphate synthases (GPPSs), and two genes encoding farnesyl pyrophosphate synthases (FPPSs). Three genes corresponded to geranyl(geranyl)pyrophosphate synthases (GGPPS1-3). In our phylogeny, GGPPS1 localised to clade V, corresponding to homomeric GGPPSs found across land plants; GGPPS2 localized to clade IV, angiosperm homomeric GPPSs; and GGPPS3 was in clade VI, which features the catalytically inactive small subunit (SSU) GGPPSs, with roles in modulating product selectivity. GGPPS1 showed the highest transcript abundance compared with the rest of the prenyltransferase genes.

When examining the gene expression across the different tissue types, an overall variability in the expression levels of all genes across the tissues was observed (Fig. 1). Across all tissues, FPPS1 was expressed at far higher levels than FPPS2. Based on tissue type there was a higher transcription level in female flowers as opposed to the other tissues. FPPS2 however showed higher transcript levels in young leaves compared to other tissue types. As both FPPS isoforms appear to have different expression patterns, it is plausible that their function and activity may differ too. To better understand the evolutionary history of FPPSs, the phylogeny of the FPPS gene family using homologous sequences collected from JGI Phytozome 12 and OneKP project databases was constructed (Table S6). The maximum likelihood phylogeny shows that the two FPPSs are from distinct subfamilies which originate from an ancient duplication in the angiosperm lineage (Figure S2), with two FPPS clades present in saxifragales, rosids, asterids, ericales and Caryophyllales, and the duplication appearing to occur after the divergence of Ranunculales.

**Fig. 3:**
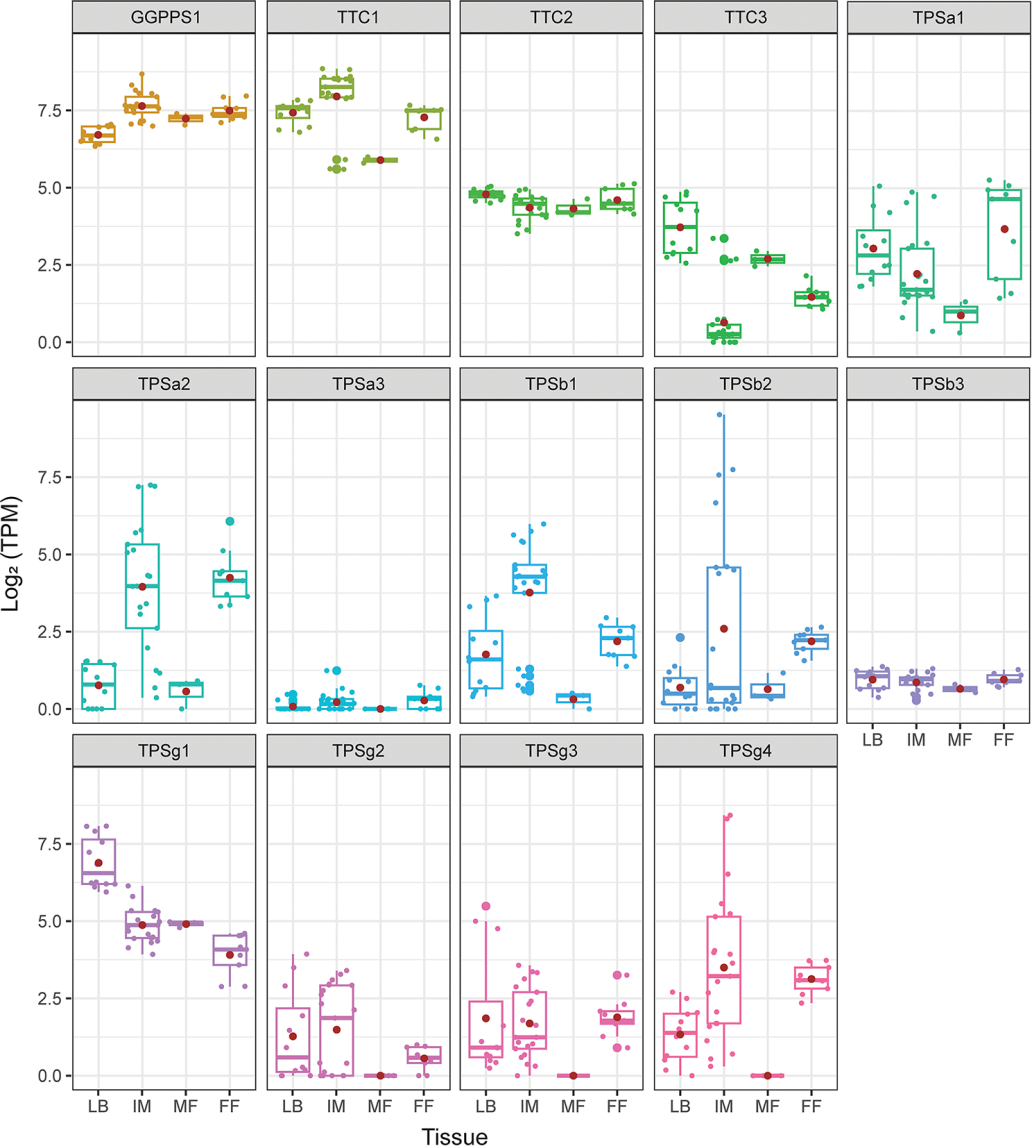
Gene expression analysis of *Daphniphyllum macropodum* TTCs, GGPPS and TPSs. Expression levels of *D. macropodum* TTCs, PTs and TPSs in the different tissue types are reported. leaf buds (LB), immature leaves (IM), male flowers (MF) and female flowers (FF)

As GGPPS1 showed the highest transcript abundance compared with the rest of the prenyltransferase genes (Fig. 3), we elected to characterize it in vivo together with geranyl linalool synthase (AtGLS) from *A. thaliana*, an enzyme known to convert GGPP to the acyclic diterpene geranyl linalool **3** (Fig. 2C). Both GGPPS1 and AtGLS were expressed in *N. benthamiana*, with either DXS or HMGR. Our results demonstrated that co-expressing HMGR, AtGLS, and GGPPS1 increased the production of geranyl linalool **3** by approximately 12-fold compared to expressing only HMGR and AtGLS (Fig. 2C and D). This confirms the GGPPS1 annotation of this gene as a *D. macropodum* GGPP synthase.

### Identification and characterisation of *D. macropodum* triterpene cyclases (TTCs)

**Fig. 4:**
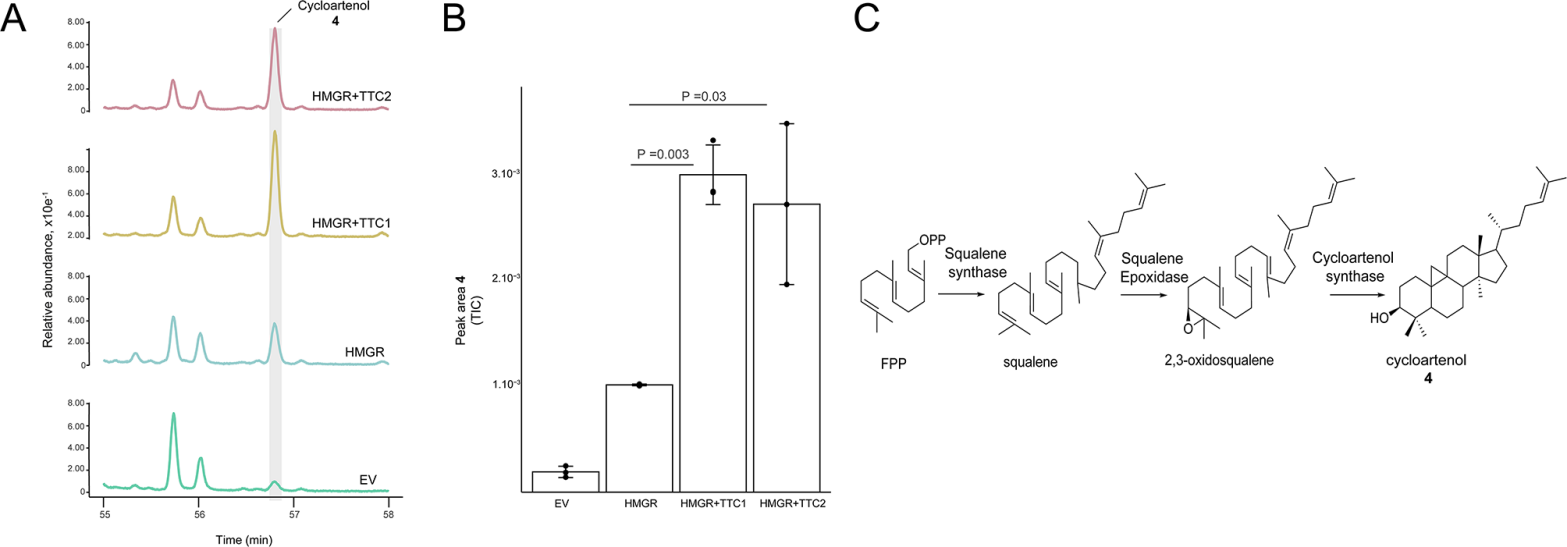
Characterisation of *Daphniphyllum macropodum* genes (TTC1, TTC2) through heterologous expression in *N. benthamiana.***(A)** GC-MS analysis of *N. benthamiana* extracts expressing the two triterpene cyclases TTC1 and TTC2 with HMGR. The GC-MS traces are presented as total ion chromatograms (TIC). Cycloartenol **4** was identified through the comparison of its mass spectrum to available spectral libraries. **(B)** Comparison of peak areas of cycloartenol in the extracts of plants expressing HMGR with TTC1 or TTC2 against HMGR. For each condition three independent biological replicates were used. data are mean ± s.d. of the three replicates. Statistical significance was assessed using a one-tailed Student’s t-test with an assumption of unequal variance. **(C)** Biosynthetic route to cycloartenol

Three *D. macropodum* genes were annotated as triterpene cyclases, namely, TTC1, TTC2 and TTC3. To determine their enzymatic activity, we expressed these genes with tHMGR in *N. benthamiana* (Table S7). The analysis of the GC-MS chromatograms showed that cycloartenol **4**, a triterpene naturally accumulating in *N. benthamiana* leaves, increased ~ 3-fold with TTC1 and TTC2 (Fig. 4, Figure S3) indicating that these two triterpene cyclases are cycloartenol synthases. No activity was determined for TTC3.

To conduct the phylogenetic analysis of TTC1 and TTC2 and TTC3 a maximum likelihood tree was constructed including the three TTCs and triterpene cyclases from other plants (Figure S4, Table S8). The phylogenetic analysis demonstrated that TTC1 and TTC2 were grouped into cycloartenol synthase clade while TTC3 was clustered with other lupeol synthases.

Regarding the expression levels of these TTCs, TTC1 exhibited the highest levels of expression compared with the other genes in all four tissues (leaf buds, immature leaves, male, and female flower) (Fig. 3).

### Identification and characterisation of *D. macropodum* terpene synthases (TPSs)

An HS-SPME-GC-MS (head space solid phase microextraction) analysis of immature leaves, flowers, and fruit tissues was performed (Figure S5) to identify *Daphniphyllum* terpenoids. The detected peaks were annotated using available MS libraries, confirming the presence of α-terpineol, linalool, and limonene in all analysed tissues.

Based on the functional annotation results of *D. macropodum* transcriptome, ten potential terpene synthases were identified. The full-length cDNAs of these genes were successfully amplified and sequenced (Table S7). A maximum likelihood phylogenetic tree of *D. macropodum* TPSs and other plant terpene synthases was generated (Figure S6, Table S9). The results showed that terpene synthases from various species were grouped into seven clades recognised in the literature (TPSa-h). All *D. macropodum* terpene synthases identified in this study were clustered into angiosperm-specific clades TPS-a, TPS-b, and TPS-g, and were named according to their clade (TPSa1, TPSa2, TPSa3; TPSb1, TPSb2, TPSb3; TPSg1, TPSg2, TPSg3, TPSg4).

The multiple sequence alignment of *D. macropodum* TPSs revealed several conserved regions essential for substrate binding and enzyme catalysis (Figure S7). These conserved regions include RRX8W, DDXX(D/E), and (N, D)DX2(S,T,G)X3E (NSE/DTE). All analysed TPSs contained the conserved DDXX(D/E) motif, while the RRX8W motif was found exclusively in TPSa1, TPSb1, TPSb2, and TPSb3.

The (N,D)DX2(S, T,G)X3E (NSE/DTE) motif was present in all aligned TPSs except TPSg3 which exhibited a slight variation. A unique amino acid substitution was observed in this region, where the expected glutamate was replaced by tyrosine. Notably, TPSg3 is shorter than typical plant TPSs and, like other TPSg clade enzymes, lacks the RRX8W motif. Additionally, its C-terminal region appeared significantly shorter. Furthermore, the alignment revealed that *D. macropodum* terpene synthases belonging to the TPS-a clade (TPSa1, TPSa2, and TPSa3) possess a shorter N-terminal extension compared to other aligned TPSs. The gene expression levels of the different TPSs were also assessed. The comparison of the expression between the different tissues for TPSs demonstrated an obvious difference in expression specifically between male and female flowers. Most TPSs seem to have higher expression in female flowers compared with male flowers.

**Fig. 5:**
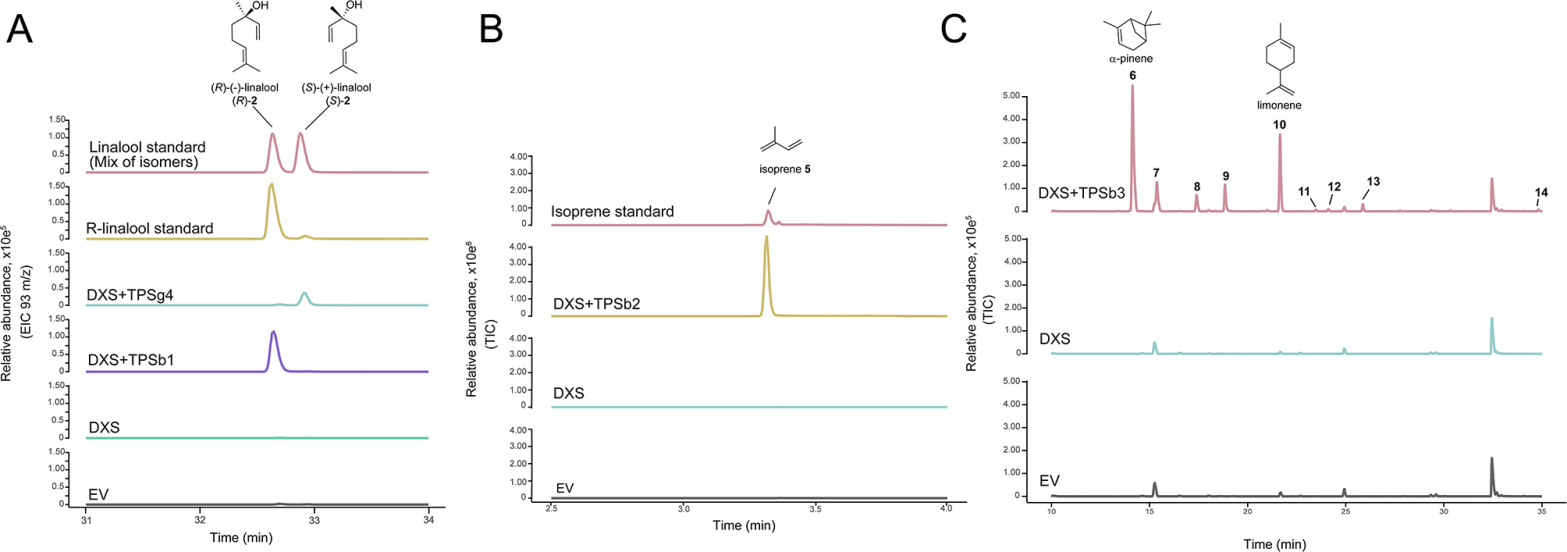
Characterisation of *Daphniphyllum macropodum* TPSs (TPSb1, TPSg4, TPSb2, and TPSb3). **(A)** HS-SPME chromatograms (chiral GC-MS separation) of *N. benthamiana* extracts expressing the TPSb1 and TPSg4 with DXS. The GC-MS traces are presented as extracted ion chromatograms (EIC 93 m/z). (*R*)-Linalool (*R*)-**2** and (*S*)-linalool (*S*)-**2** forms were confirmed through comparison with available authentic standards. **(B)** HS-SPME chromatograms of *N. benthamiana* extracts expressing the TPSb2 with DXS. The GC-MS traces are presented as total ion chromatograms. Isoprene **5** was identified through comparison with its authentic standard. **(C)** HS-SPME chromatograms of *N. benthamiana* extracts expressing the TPSb3 with DXS. The GC-MS traces are presented as total ion chromatograms. Peak representing α-pinene **6** and limonene **10** were identified based on comparisons with authentic standards (Figure S9). The rest of the peaks were annotated based on similarities with the available spectral libraries (Table S11): unknown **7**, *p*-mentha-1(7),8-diene **8**, β-pinene **9**, unknown **11**, unknown **12**, α-terpinolene **13**, and unknown **14**

Next, the ten uncharacterised and amplified TPS enzymes were functionally tested by expression in *N. benthamiana* through co-expression with the rate-limiting enzymes DXS and/or HMGR (Table S4). The overexpression assay in *N. benthamiana* of TPSb1 led to the production of (*R*)-linalool (*R*)-**2**, and the expression of TPSg4 led to the production of (*S*)-linalool (*S*)-**2**. The two isomers were confirmed by comparison with authentic standards using chiral HS-SPME method (Fig. 5A). The phylogenetic tree analysis demonstrated that TPSb1 and TPSg4 belong to two distinct clades TPSb and TPSg respectively (Figure S6).

In order to verify whether these enantiomers accumulate in the plant, the volatile emissions of *D. macropodum* leaves and fruits were examined using the same chiral HS-SPME method (Figure S8). The analysis indicated that both forms of linalool, (*R*)-linalool [(*R*)-**2**] and (*S*)-linalool [(*S*)-**2**], were present. The analysis of expression patterns across tissues for TPSb1 and TPSg4 genes, showed noticeable similarity in the expression both in terms of overall abundance and of abundance in specific tissues (Fig. 3).

All the remaining TPSs were assessed for activity as described in the experimental procedures. For instance, the expression of DXS with TPSb2 in *N. benthamiana* resulted in a product that was identified as isoprene **5** based on comparison with an authentic standard (Fig. 5B). Next, the expression of DXS with TPSb3 resulted in the production of a blend of monoterpenes that were detected with the HS-SPME method. These monoterpenes included monocyclic structures (limonene **10**, *p*-mentha-1(7),8-diene **8**, α-terpinolene **13**) and bicyclic structures (α-pinene **6** and β-pinene **9**). α-pinene **6** and limonene **10** were identified with comparison to authentic standards. *p*-mentha-1(7),8-diene **8**, β-pinene **9** and α-terpinolene **13** were identified based on MS-libraries comparisons. Additional products were detected and labelled as unknown (Fig. 5C, Figure S9).

**Fig. 6:**
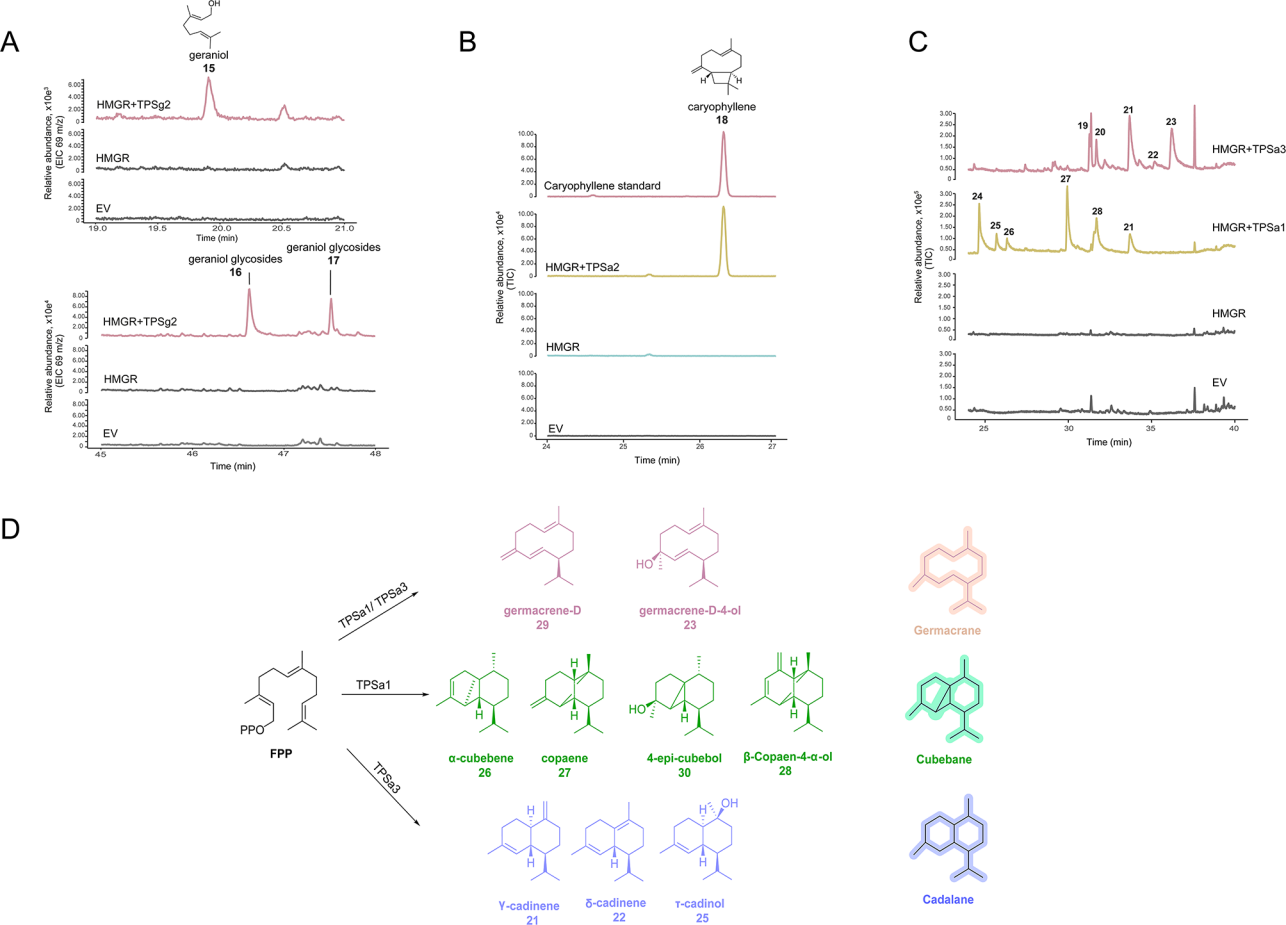
Characterisation of *D. macropodum* TPSs through heterologous expression in *N. benthamiana.***(A)** GC-MS chromatograms of *N. benthamiana* leaf extracts expressing TPSg2 showing as products: geraniol **15** and geraniol glycosides **16**, **17**. **(B)** GC-MS chromatograms of *Nicotiana benthamiana* leaf extracts expressing TPSa2 showing caryophyllene **18** as a main product. **(C)** GC-MS chromatograms for TPSa1 and TPSa3. Peak IDs are as follows, for TPSa3: γ-cadinene **19**; δ-cadinene **20**; germacrene D-ol **21**; unknown **22**; τ-cadinol **23**. For TPSa1: α-cubebene **24**; copaene **25**; β-copaen-4-α-ol **26**; germacrene D **27**; 4-epi-cubebol **28**. **(D)** Chemical structures for TPSa1 and TPSa3 products

Further TPSs were characterised, the overexpression of TPSg3 with DXS in *N. benthamiana* and the GC-MS analysis of the corresponding plant extracts showed that TPSg3 catalyses the formation of a sesquiterpene product that was identified as α-guaiene through spectral library matches (Figure S10). *N. benthamiana* plants expressing HMGR and TPSg2 showed GC-MS chromatograms with products identified as geraniol **15** and geraniol glycosides **16** (based on comparison with available spectral libraries). It has been reported previously that geraniol **15** accumulates predominantly as geraniol glycosides when expressed *in planta* due to the activity of endogenous enzymes [22, 23] (Fig. 6A, Figure S11).

Similarly, when TPSa2 was co-expressed with HMGR in *N. benthamiana*, the formation of a product identified as caryophyllene **18** was detected. This chemical was confirmed by comparison with an authentic standard (Fig. 6B). Two additional terpene synthases were assessed for activity both in vivo and in vitro; namely TPSa1 and TPSa3. Their expression in *N. benthamiana* with HMGR demonstrated that both enzymes catalyse the formation of a blend of sesquiterpene products. The products were mostly identified based on comparisons with the available MS-libraries (Table S10 and S11).

The identified products of TPSa1 were sesquiterpenes with a germacrane scaffold, including germacrene D **27**, germacrene D-ol **21**, and those with a cubebane scaffold, such as α-cubebene **24**, copaene **25**, β-copaen-4-α-ol **26**, and 4-epi-cubebol **28**. Meanwhile, the products of TPSa3 comprised sesquiterpenes with a cadalane scaffold, namely γ-cadinene **19**, δ-cadinene **20**, and τ-cadinol **23** (Fig. 6C/D). The enzymatic characterisation of these two enzymes was also performed in vitro through an enzymatic assay with sesquiterpene precursor FPP. The GC-MS results showed that both enzymes were also active in vitro however only some of the major products detected *in planta* were also detected in vitro (Figure S12). For TPSa1 we detected germacrene D and germacrene D-ol, and for TPSa3 we detected δ-cadinene and germacrene D-ol.

## Discussion

### MVA and MEP pathway genes

From the transcriptome of *D. macropodum*, we identified and analysed the expression levels of genes involved in MVA and MEP pathways. Multiple paralogs were observed for many steps in these biosynthetic pathways. For instance, in *D. macropodum* two isoforms of FPPS with distinct expression patterns were identified. Paralogous genes often play distinct roles in plant evolution and fitness, contributing to the organism’s adaptability. For instance, among the duplicated DXS copies in plants’ species such as *Arabidopsis thaliana* [24] and *Medicago truncatula* [25], only one copy has retained the ancestral function of catalysing the first step of the MEP-pathway. This alludes to a possible loss of activity or neo-functionalization of the other paralogs [26]. However, paralogs can also be functionally redundant, for example, it has been revealed that the bulk of GGPP production in tomato plastids relies on two cooperating GGPP synthase paralogs [27]. For each MVA/MEP gene in *D. macropodum* the expression of the different paralogs varied in the examined tissues which suggests variability in expression conditions and therefore functional diversity.

### TTC and TPS content

In this study, we identified three TTCs and ten TPSs. This is lower than may be expected from plant genomes, with tomato, for example, having 34 full-length TPS genes and 18 TPS pseudogenes [28], and rice having 44 TPS genes in the reference genome [29]. Our lower number is likely due to the fact we are using a *de novo* transcriptome and not a genome assembly. This means that we are missing genes with low or no expression—especially likely as we have used a limited set of tissues. Furthermore, although we have used long-reads to improve the assembly, there remains a risk of collapsed paralogs, which would reduce the number of genes. We are working towards obtaining a genome assembly of *D. macropodum* where higher numbers of TPSs may be revealed.

One of our hypotheses leading into this work was that understanding terpenoid biosynthesis in *D. macropodum* would reveal insights into the squalene derived *Daphniphyllum* alkaloid biosynthetic pathway, which we know very little about [4]. Perhaps, for example, a TTC would form a novel triterpenoid skeleton which may link terpenoid and alkaloid biosynthesis. This hypothesis was not validated: we discovered neither a TTC nor a TPS that had products clearly leading towards alkaloid biosynthesis. Therefore, the connection between terpenoids and alkaloids in *D. macropodum* remains unresolved and further investigation is required to resolve the genetic or enzymatic connection between these pathways.

### *D. macropodum* Linalool synthases

Overexpressing TPSb1 in *N. benthamiana* produced (*R*)-linalool, whereas overexpressing TPSg4 resulted in the production of (*S*)-linalool. Similar result has been shown in previous reports where plants have two terpene synthases producing the two linalool enantiomers such as *Arabidopsis thaliana* [30] and *Camellia sinensis* [31]. Phylogenetically, TPSb1 and TPSg4 are not closely related paralogs but instead fall into distinct clades of TPS proteins. Consequently, their ability to produce linalool likely evolved independently [32].

The analysis of the *D. macropodum* leaves and fruits volatiles with HS-SPME confirmed the emission of the two forms of linalool in the plant. It has previously been reported that the ecological functions of (*R*)-linalool (*R*)-**2** and (*S*)-linalool (*S*)-**2** in plants differ with (*R*)-linalool exhibiting repellent activity against various insect species [33], and (*S*)-linalool exhibiting both attractive and repellent activities [34]. These distinct functions highlight the importance of enantiopure linalool in ecological interactions context. Information on the pollination method of *D. macropodum* plants is scarce, but some authors have proposed that wind pollination may be involved [35, 36]. This suggests that the role of linalool isomers in *Daphniphyllum* is likely related to defence and/or adaptation to the environment.

### Multiproduct enzymes: TPSb3, TPSa1 and TPSa3

The three characterised enzymes TPSa1, TPSa3 and TPSb3 were multiproduct enzymes. Many terpene synthases are known to be multiproduct enzymes with nearly half of the identified mono- and sesquiterpene synthases found to have the ability to generate multiple chemicals [37]. For sessile organisms like plants, multiproduct terpene synthases offer a significant ecological advantage. They enable plants to enhance their chemical defences in a cost-effective manner and maximise the diversity and structure of compounds available for adaptation [38]. In addition, the low expression of these three enzymes in the investigated plant tissues was quite intriguing. It could be that the expression of these TPSs is usually induced only under specific environmental/ecological conditions therefore their expression was minimal in the analysed tissues.

### Sesquiterpene synthases with diverse product types: TPSa1 and TPSa3

When expressed in *N. benthamiana*, TPSa1 GC-MS traces showed as products germacrene D **27**, α-cubebene **24**, copaene **25**, germacrene D-ol **21**, β-copaen-4-α-ol **26**, and 4-epi-cubebol **28**. The ratio of sesquiterpene alcohols to sesquiterpenes was 40–60% of total products both in vivo and in vitro. It is worth noting though that germacrene D **27** is known to undergo rearrangements in acidic conditions to molecules of the cadalane and cubebane types such as cadinene **19**/**20**, copaene **25**, and cubebene **24** [39]. Despite this, the presence of alcohol sesquiterpenes as products such as germacrene D-ol **21**, β-copaen-4-α-ol **26**, and 4-epi-cubebol **28** shows the catalytic ability of this enzyme as a multiproduct TPS.

When expressed in *N. benthamiana*, TPSa3 produced γ-cadinene **19**, δ-cadinene **20**, germacrene D-ol **21** and τ-cadinol **23**. The ratio of sesquiterpene alcohols to sesquiterpenes was 90–10% in vitro and 70–30% in vivo which indicates that TPSa3 is primarily a sesquiterpene alcohol synthase. The major product of TPSa3 both in vivo and in vitro was germacrene D-ol **21**. Only a few germacrene D-ol synthases have been characterised from plant species, including *Setaria italica* [40], *Santalum album* [41], and *Taiwania cryptomerioides* [42]. Additionally, a (+)-δ-cadinene synthase from *Gossypium arboreum* was transformed into a germacrene D-ol synthase producing primarily germacrene D-ol **21** through a single residue mutation (W279A) [43, 44]. Identifying TPSa3 as a germacrene D-ol synthase opens the door for further mechanistic investigations on plant terpene alcohol synthases to pinpoint the specific residues that influence product outcomes.

## Conclusion

Our study presents the transcriptome-based discovery of TPS family genes in *D. macropodum*. We identified and characterised eleven new TPS enzymes, including sesquiTPSs, monoTPSs, and triTPSs. These enzymes demonstrated versatile biochemical functions, potentially linked to their diverse roles in ecological adaptation. Our findings offer a valuable reference for future research on terpene biosynthesis in *Daphniphyllum* *sp.*.

## Electronic supplementary material

Below is the link to the electronic supplementary material.

Supplementary Material 1

Supplementary Material 2

## Data Availability

The datasets generated during the current study are available in the NCBI repository (BioProject: PRJNA1067848). Raw reads are available on the Sequence Read Archive (SRX23355835-SRX23355841, SRR31514991-SRR31515028, SRR31543159). Sequences cloned and analysed are available on NCBI GenBank (PQ655046-PQ655059). The assembled transcriptome is available on FigShare (10.6084/m9.figshare.27913764.v1).
