# Supplementary material for: Discovery and characterisation of terpenoid biosynthesis enzymes from Daphniphyllum macropodum
Source: BMC Plant Biol. 2025 Apr 16;25:483. doi: 10.1186/s12870-025-06421-0 (PMC12001401; doi:10.1186/s12870-025-06421-0)
Supplement: Supplementary file 2 — Supplementary Material 2 [file 12870_2025_6421_MOESM2_ESM.pdf]

Supplementary information for

Discovery and characterisation of terpenoid biosynthesis enzymes from *Daphniphyllum macropodum*

Kaouthar Eljounaidi[1,3], Caragh B. Whitehead[1], Emily Radley[1], Marissa H. Petrou[1], Katherine Newling[2], Sally James[2] and Benjamin R. Lichman[1]

Affiliations

[1] Centre for Novel Agricultural Products, Department of Biology, University of York, York, YO10 5DD, UK
[2] Biosciences Technology Facility, Department of Biology, University of York, York, UK
[3] Current address: Manchester Institute of Biotechnology and Department of Chemistry, The University of Manchester, 131 Princess Street, Manchester, M1 7DN

Contents

Supplementary Figures S1 to S12

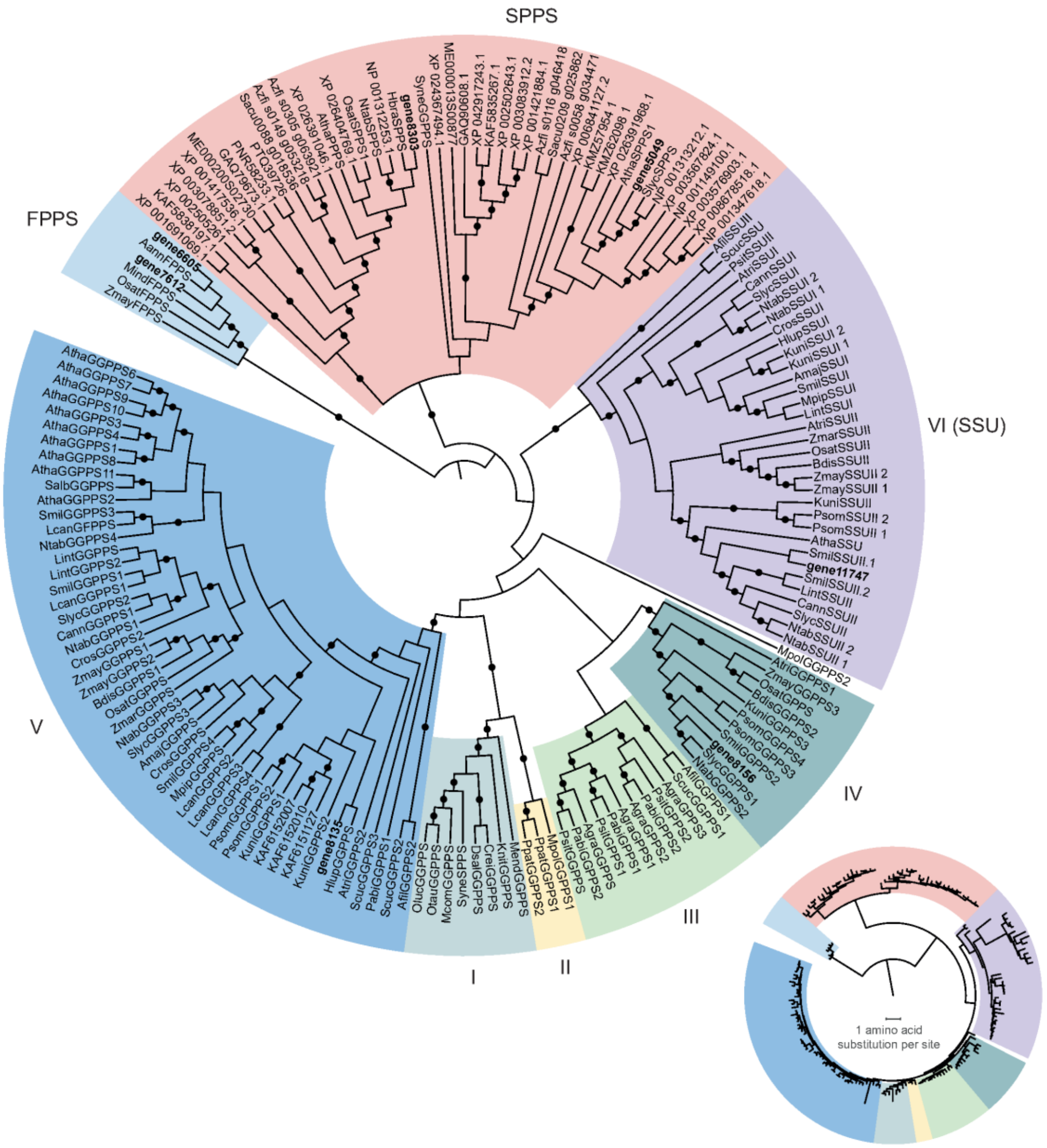

Figure S1. Evolution of geranyl(geranyl) pyrophosphate synthases (G[G]PPSs) in plants. Maximum likelihood phylogenetic tree (cladogram) of plant G(G)PPSs, with phylogram inset (with branch lengths). Major clades are highlighted with different colours, and labelled as described by Song *et al* [1]. Tree rooted on farnesyl pyrophosphate synthase (FPPS) clade (see Figure S2 below). Clade I = algal GGPPSs; II = byrophyte GGPPSs; III = fern/gymnosperm homomeric GGPPS/GPPS; IV = angiosperm homomeric GPPSs; V = GGPPSs; VI = small subunits (SSU, selectivity modifiers). SPPS = solanesyl pyrophosphate synthases. Sequences from *D. macropodum* highlighted in bold. Circles show branches with >85% and >95% support as judged by 1000X SH-aLRT and UltraFast Boostrapping replicates, respectively.

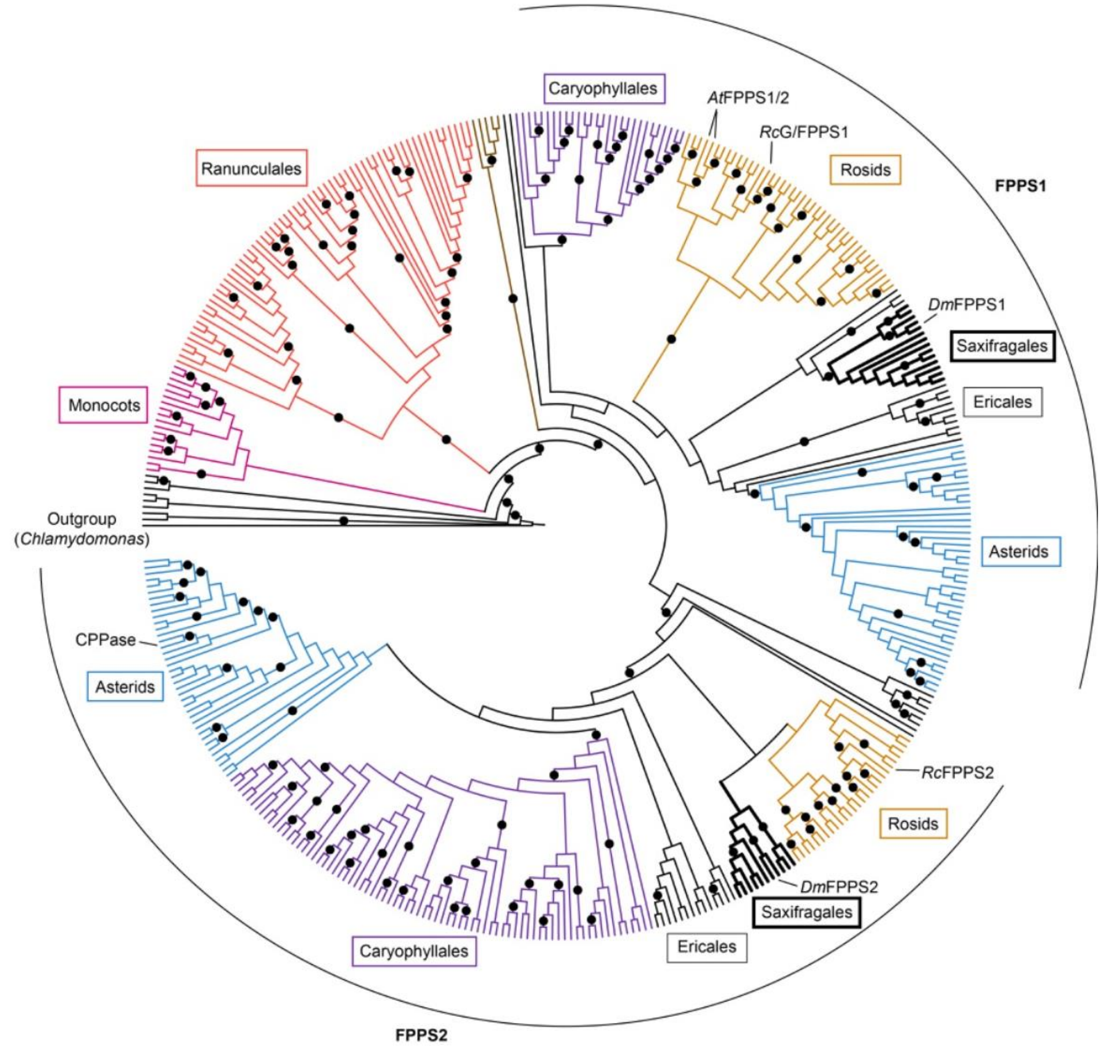

Figure S2. Evolution of farnesyl pyrophosphate synthase (FPPS) in plants. Maximum likelihood phylogenetic tree (cladogram) of plant FPPSs. Major clades are highlighted with different colours, the branches from Saxifragales highlighted in bold. Circles show branches with >85% and >95% support as judged by 1000X SH-aLRT and UltraFast Boostrapping replicates, respectively.. Chlamydomonas was used as an outgroup.

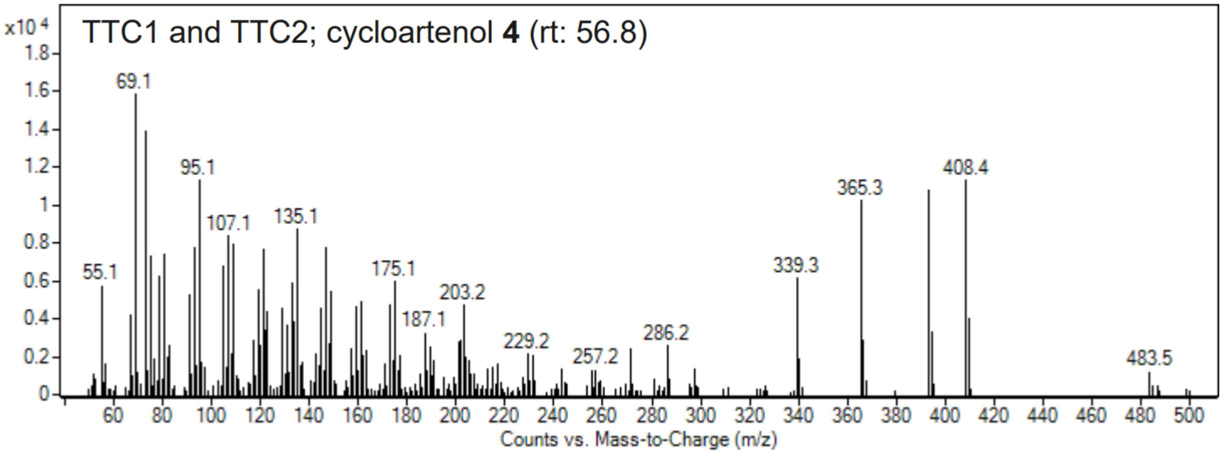

Figure S3. Triterpene formation. Electron impact spectrum of TTC1/TTC2 product, cycloartenol **4**.

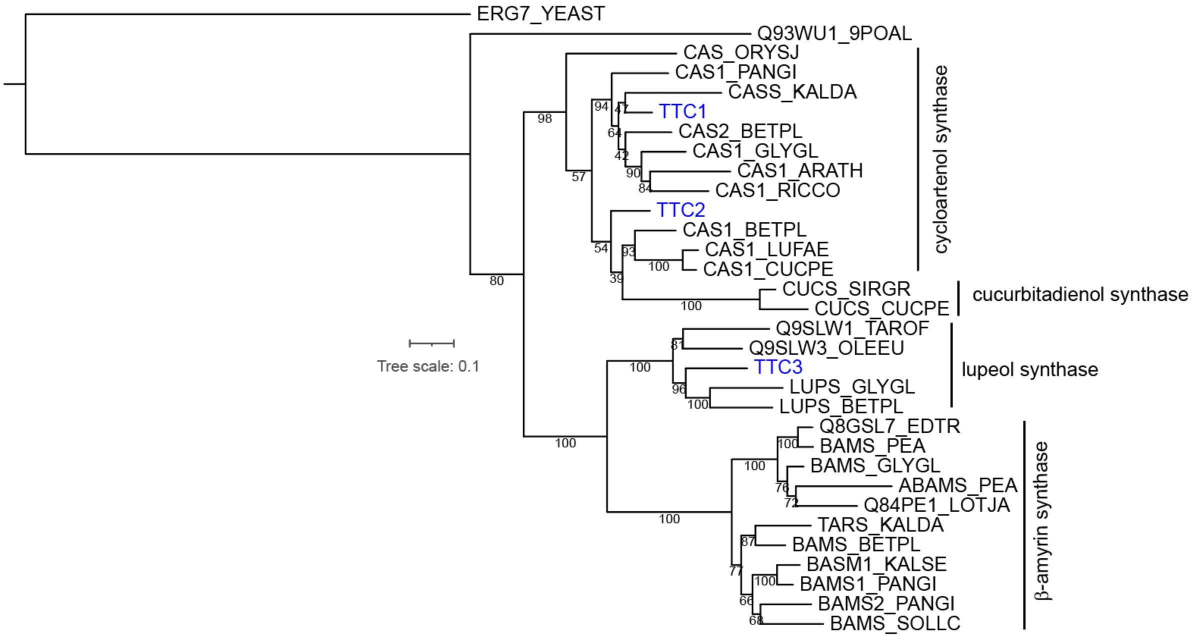

Figure S4. Phylogenetic analysis of triterpene cyclases (TTCs). Included are *Daphniphyllum macropodum* triterpene synthases TTC1, TTC2 and TTC3 with triterpene synthases from other plants. The scale of the maximum likelihood tree is one amino acid substitution per site. The text on the branches are support values (UFBoot). *D. macropodum* TTCs are marked in blue.

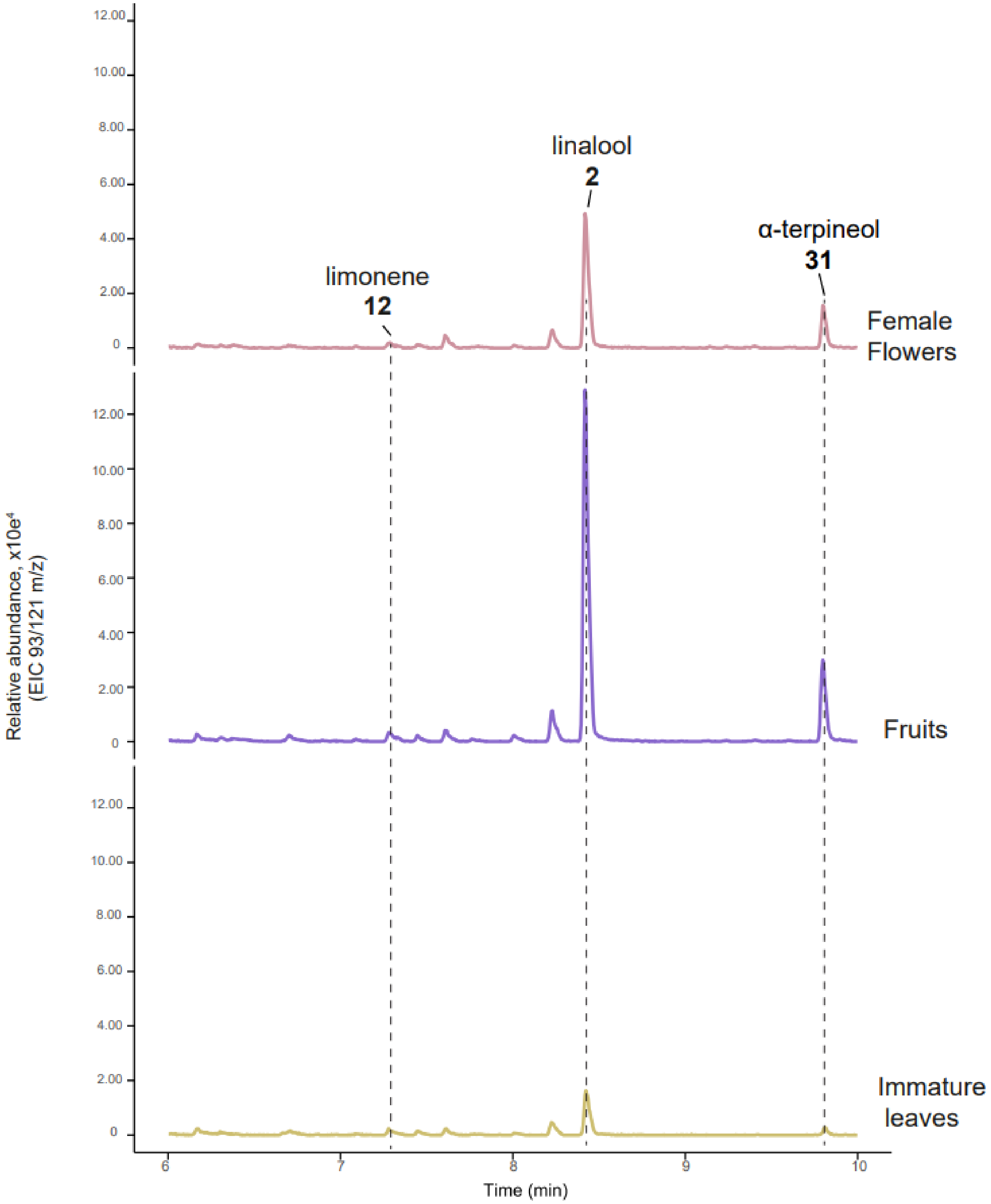

Figure S5. Volatile analysis of *D. macropodum*. HS-SPME-GC-MS chromatograms of *D. macropodum* immature leaves, flowers, and fruits, illustrating the accumulation of limonene **12**, linalool **2**, and α-terpineol **31** in the plant.

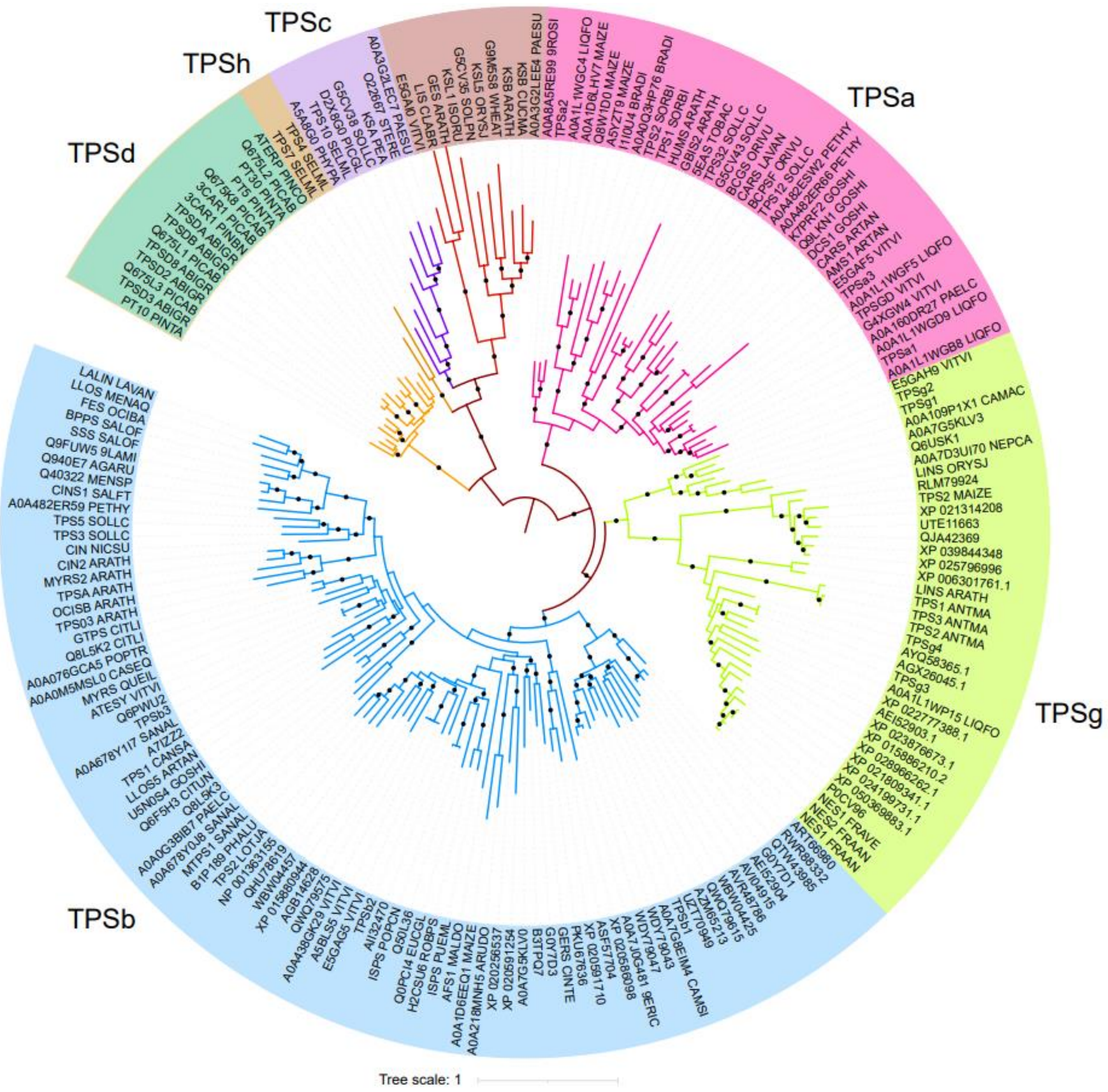

Figure S6. **Phylogenetic analysis of plant terpene synthases (TPSs).** Maximum-likelihood phylogenetic analysis of *Daphniphyllum macropodum* TPSs together with other characterised plant terpene synthases. The scale measures evolutionary distances in amino acid substitutions per site. *Dapniphyllum* genes are in bold. Circles show branches with >95% support as judged by UltraFast Boostraps.

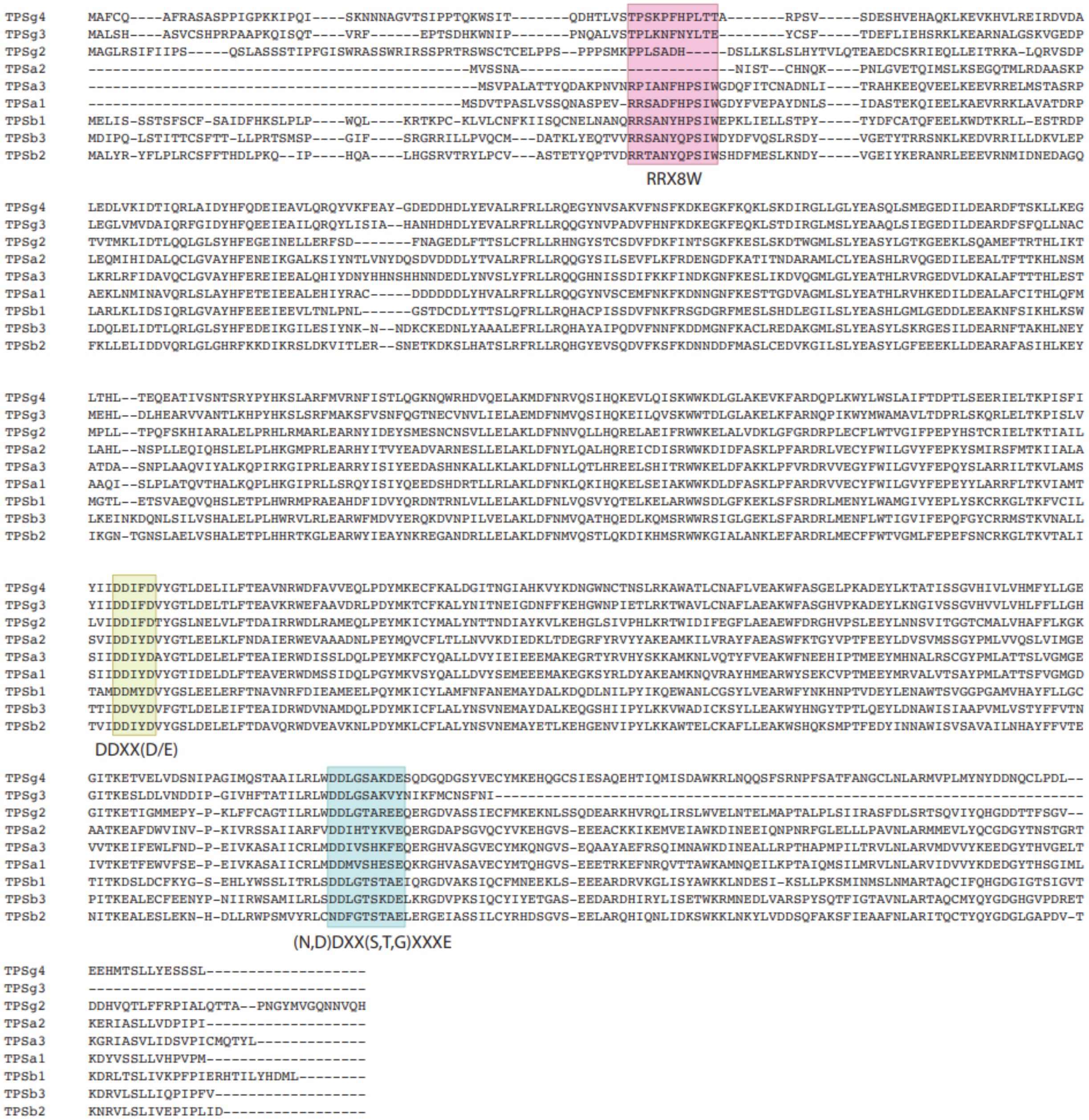

Figure S7. Alignment of TPSs. Multiple sequence alignment of TPSs with conserved regions highlighted.

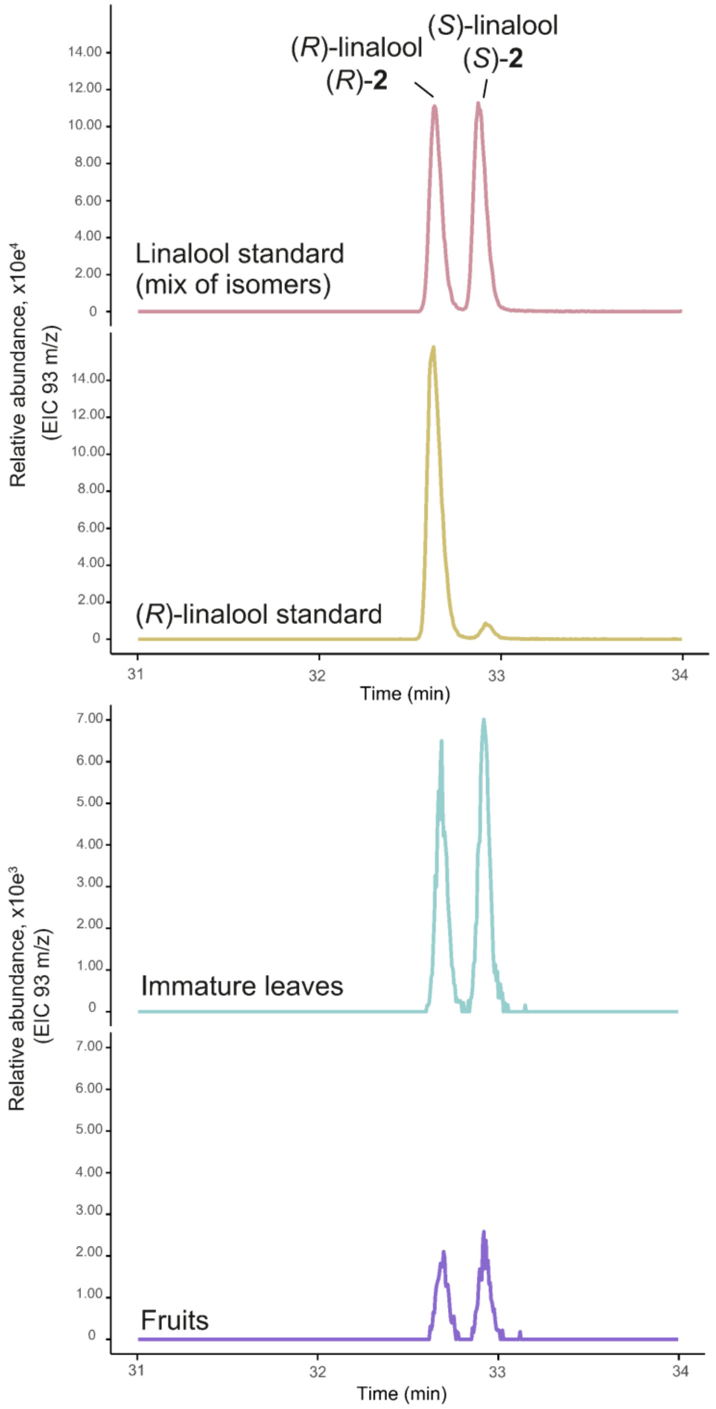

Figure S8. Accumulation of (*R*)-linalool and (*S*)-linalool in *Daphniphyllum* tissues. HS-SPME chromatograms of *D. macropodum* leaves and fruits showing the accumulation of both isomers of linalool **2** in the plant.

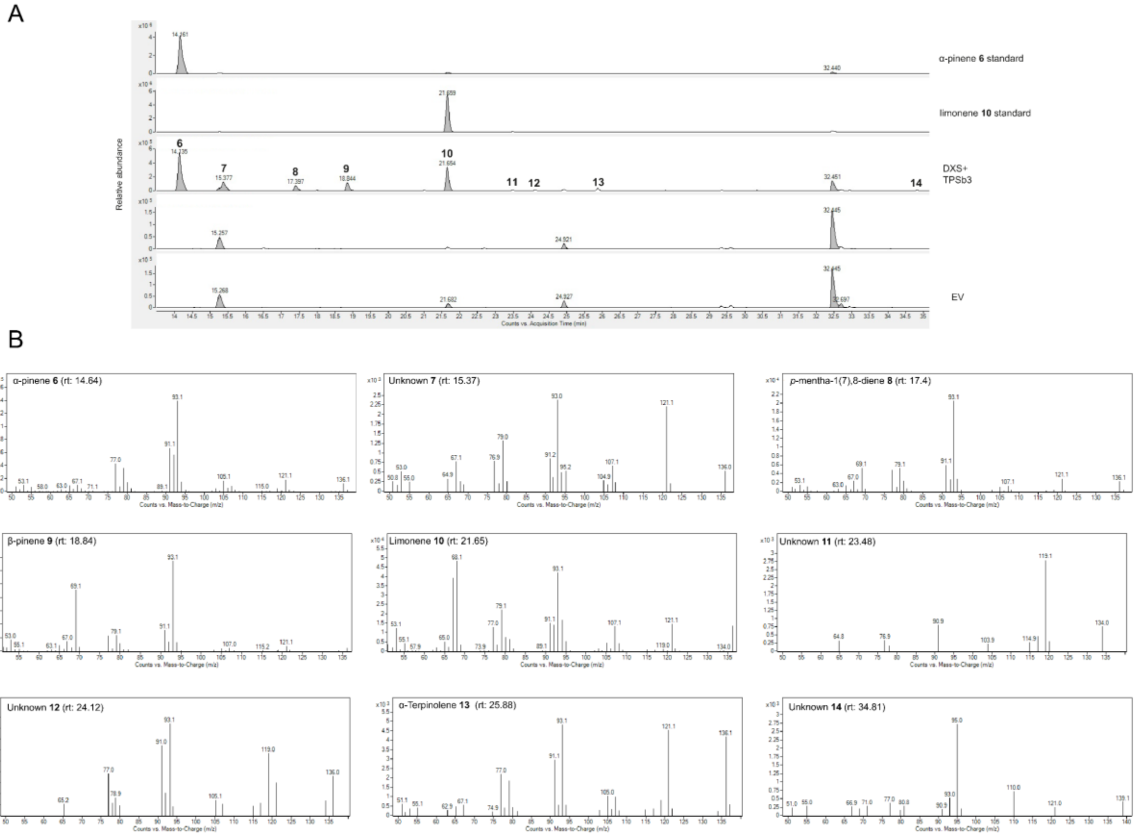

Figure S9. Characterisation of TPSb3 through heterologous expression in *N. benthamiana.* **A.** HS-SPME chromatograms of *N. benthamiana* leaf extracts expressing TPSb3 showing nine different monoterpene products. **B.** Electron impact mass spectra of the detected peaks.

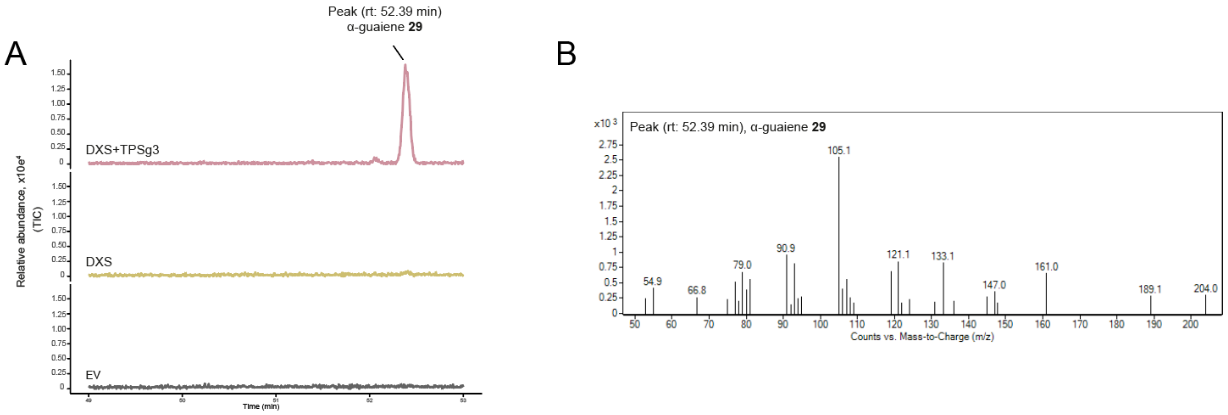

Figure S10. Characterisation of TPSg3 in vivo through heterologous expression in *N. benthamiana.* **A.** Chromatograms of *N. benthamiana* leaf extracts expressing TPSg3 showing a peak at min 52.39. **B.** MS spectrum of the product at min 52.39 annotated as α-guaiene **29**.

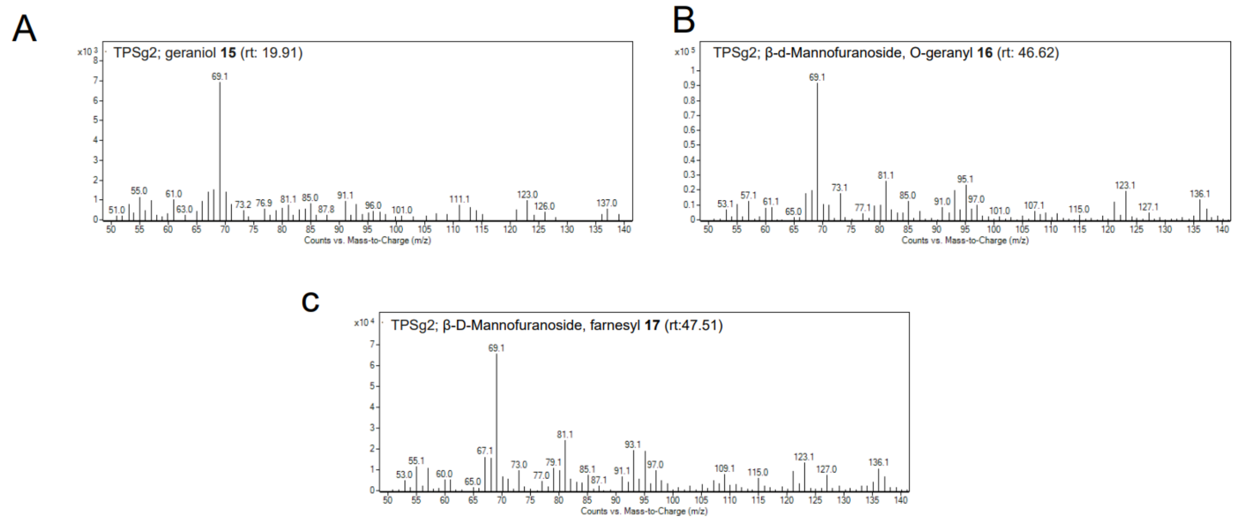

Figure S11. Mass spectra for TPSg2 products. TPSg2 products: **A.** geraniol **15**. **B.** O-geranyl β-d-mannofuranoside **16**, and **C.** farnesyl β-D-Mannofuranoside **17**.

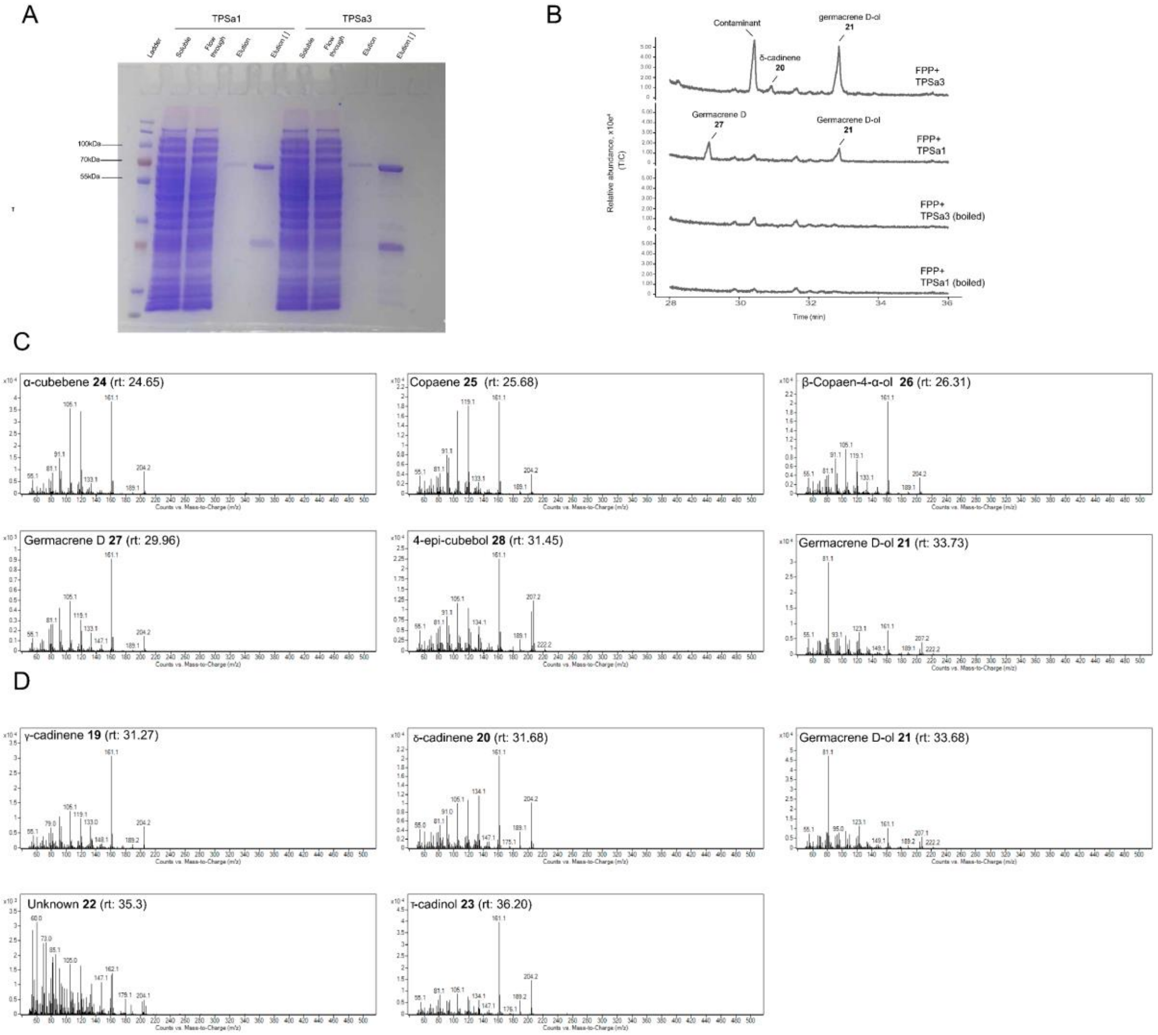

Figure S12. Confirmation of TPSa1 and TPSa3 functions with purified protein. A. TPSa1 and TPSa3 were each expressed as 6xHis N-terminally tagged proteins in *E. coli* BL21 cells and purified via Ni-NTA affinity chromatography. SDS-PAGE gel of fractions from their purification with Coomassie blue staining are shown. Fractions are as follows: Soluble fraction, flow through, eluted protein before concentrating, eluted protein after concentrating. **B.** GC-MS chromatograms from the in vitro reactions of TPSa1 and TPSa3 with FPP as substrate. TIC chromatograms of negative controls (boiled proteins) are also shown for comparisons. **C.** The MS spectra of all the peaks detected as TPSa1 products are shown (including the peaks observed through the in vivo experiments). **D.** MS spectra the peaks detected as TPSa3 products.

Reference

1. Song S, Jin R, Chen Y, He S, Li K, Tang Q, et al. The functional evolution of architecturally different plant geranyl diphosphate synthases from geranylgeranyl diphosphate synthase. Plant Cell. 2023;35:2293–315.
